# Dextran sulfate prevents excess aggregation of human pluripotent stem cells in 3D culture by inhibiting ICAM1 expression coupled with down-regulating E-cadherin through activating the Wnt signaling pathway

**DOI:** 10.1186/s13287-022-02890-4

**Published:** 2022-05-26

**Authors:** Haibin Wu, Xianglian Tang, Yiyu Wang, Ning Wang, Qicong Chen, Jinghe Xie, Shoupei Liu, Zhiyong Zhong, Yaqi Qiu, Ping Situ, Mark A. Zern, Jue Wang, Honglin Chen, Yuyou Duan

**Affiliations:** 1 Laboratory of Stem Cells and Translational Medicine, Institutes for Life Sciences, School of Medicine, South China University of Technology, No. 382 Waihuan East Road, Suite 406, Higher Education Mega Center, Guangzhou, 510006 People’s Republic of China; 2 School of Biomedical Sciences and Engineering, South China University of Technology, Guangzhou International Campus, Guangzhou, 510180 People’s Republic of China; 3 Guangxi Key Laboratory of Reproductive Health and Birth Defects Prevention, Guangxi Health Commission Key Laboratory of Precise Diagnosis and Treatment of Genetic Diseases, Maternal and Child Health Hospital of Guangxi Zhuang Autonomous Region, Nanning, 530003 Guangxi People’s Republic of China; 4 Genetic and Metabolic Central Laboratory, Guangxi Birth Defects Research and Prevention Institute, Nanning, 530003 Guangxi People’s Republic of China; 5 Department of Internal Medicine, University of California Davis Medical Center, Sacramento, CA 95817 USA; 6 National Engineering Research Center for Tissue Restoration and Reconstruction, South China University of Technology, Guangzhou, 510180 People’s Republic of China; 7 Guangdong Provincial Key Laboratory of Biomedical Engineering, South China University of Technology, Guangzhou, 510180 People’s Republic of China; 8 Key Laboratory of Biomedical Materials and Engineering of the Ministry of Education, South China University of Technology, Guangzhou, 510180 People’s Republic of China

**Keywords:** Human pluripotent stem cells, Suspension culture, Cell aggregates, Dextran sulfate, Cellular adhesion molecules

## Abstract

**Background:**

Human pluripotent stem cells (hPSCs) have great potential in applications for regenerative medicine and drug development. However, 3D suspension culture systems for clinical-grade hPSC large-scale production have been a major challenge. Accumulating evidence has demonstrated that the addition of dextran sulfate (DS) could prevent excessive adhesion of hPSCs from forming larger aggregates in 3D suspension culture. However, the signaling and molecular mechanisms underlying this phenomenon remain elusive.

**Methods:**

By using a cell aggregate culture assay and separating big and small aggregates in suspension culture systems, the potential mechanism and downstream target genes of DS were investigated by mRNA sequence analysis, qRT-PCR validation, colony formation assay, and interference assay.

**Results:**

Since cellular adhesion molecules (CAMs) play important roles in hPSC adhesion and aggregation, we assumed that DS might prevent excess adhesion through affecting the expression of CAMs in hPSCs. As expected, after DS treatment, we found that the expression of CAMs was significantly down-regulated, especially E-cadherin (E-cad) and intercellular adhesion molecule 1 (ICAM1), two highly expressed CAMs in hPSCs. The role of E-cad in the adhesion of hPSCs has been widely investigated, but the function of ICAM1 in hPSCs is hardly understood. In the present study, we demonstrated that ICAM1 exhibited the capacity to promote the adhesion in hPSCs, and this adhesion was suppressed by the treatment with DS. Furthermore, transcriptomic analysis of RNA-seq revealed that DS treatment up-regulated genes related to Wnt signaling resulting in the activation of Wnt signaling in which SLUG, TWIST, and MMP3/7 were highly expressed, and further inhibited the expression of E-cad.

**Conclusion:**

Our results demonstrated that DS played an important role in controlling the size of hPSC aggregates in 3D suspension culture by inhibiting the expression of ICAM1 coupled with the down-regulation of E-cad through the activation of the Wnt signaling pathway. These results represent a significant step toward developing the expansion of hPSCs under 3D suspension condition in large-scale cultures.

**Supplementary Information:**

The online version contains supplementary material available at 10.1186/s13287-022-02890-4.

## Background

Human pluripotent stem cells (hPSCs), including human embryonic stem cells (hESCs) [1] and human-induced pluripotent stem cells (hiPSCs) [2], have great potential as a robust cell source in regenerative medicine due to their capacity for self-renewal and multi-lineage differentiation potential [3]. In most previous studies, hPSCs were conventionally cultured in a two-dimensional (2D) adherent condition, allowing not only long-term expansion but also maintenance of hPSCs with high quality. However, this approach is highly dependent on feeder cells or commercial matrices [4, 5]. Moreover, 2D culture of hPSCs occupies a large space and consumes too much effort for scaling up production, for example, by using a multilayered cell factory [6], thus impeding the application of hPSCs in clinical treatment because of the difficulty in obtaining a large number of high-quality stem cells. For example, in clinical applications of cell-based therapies, at least one billion functional cells are required for each patient to restore the function of a damaged organ such as the liver [7], pancreas [8], or heart [9]. In order to solve this problem, three-dimensional (3D) suspension culture combined with bioreactors has been developed for large-scale production and integrated differentiation of hPSCs.

There are several kinds of 3D culture methods that have been established for large-scale expansion of hPSCs, including cell aggregates [10, 11], cells on microcarriers [12], and cells embedded in microcapsules [13]. Among these approaches, expansion of hPSCs in an aggregate form has been widely employed, because it is not only independent of biological materials or matrices (e.g., Matrigel) from animal origins, but it is also much easier to retrieve cells, making it more convenient than microcarrier/microcapsule-based approaches. By using cell aggregate approaches, researchers have achieved a yield of up to 1.5 × 10^6^ hPSCs per milliliter with good maintenance of pluripotency, and the ability for long-term, good manufacturing practice (GMP) grade serial expansion of hPSCs [14]. Moreover, the expansion of hPSCs as aggregates under 3D suspension culture systems is also adaptive to different bioreactors, such as static bag [14], dynamic spinner [15], horizontal stirred bioreactor [16], and vertical-wheel bioreactor [17]. This suggests that the use of a suspension culture system with aggregates may be a powerful and promising method for the large-scale production of hPSCs.

However, the size of aggregates is a crucial parameter for hPSC doubling, retrieving and the integrated differentiation process into functional somatic cells [18]. The excessive aggregation of hPSCs in 3D suspension culture conditions hampers the differentiation process. Farzaneh et al*.* investigated the size of hPSC aggregates on their capacity for differentiating into definitive endoderm spheres, and they found that bigger aggregates resulted in a lower SOX17 positivity rate [19]. In addition, apoptosis is more likely to happen in cell aggregates with bigger sizes rather than those with smaller sizes in our study and others [20]. This may be due to the limited ability of nutrients and oxygen to diffuse toward the interior of the bigger hPSC aggregates, leading to the generation of hypoxia and necrosis in the central of aggregates [21–23]. Therefore, it is important to control the size of aggregates in developing repeatable and high-performance hPSC large-scale production.

Researchers have attempted several physical and biochemical approaches to produce size-controlled and homogeneous hPSC aggregates under suspension culture conditions. One simple approach to obtain the size-controlled hPSC aggregates was by seeding a determined number of cells into microwell culture plates [24]; however, this method could not be combined with bioreactor suspension culture systems and was limited for hPSC large-scale expansion. The most reported approach was utilizing different agitation rates of spinner flasks or bioreactors to suppress excessive aggregation of hPSCs [25, 26]. Employing impeller stirring ensured the homogeneous distribution of nutrients and gases in suspension culture. The stirred-type bioreactor has been extensively used in manufacturing traditional biological products, which emphasized the increased production of the proteins, such as recombination proteins generated by Chinese hamster ovary (CHO) cells, rather than the quality of cells [27, 28]. However, the purpose of hPSC large-scale expansion is to harvest high-quantity and high-quality stem cells and overly high shear stress has a negative effect on cell viability and differentiation [29–31]. Thus, bioreactors which control aggregate size by operating at a high rotational speed may not constitute the best solution for hPSC suspension culture. A better approach may be to avoid excessive adhesion between aggregates by adding chemical reagents, such as methylcellulose [14], knockout serum replacement (KSR) [32], lysophosphatidic acid [33], sphingosine-1-phosphate [33], or dextran sulfate (DS) [34]. Among these chemical reagents, DS, a polysulfated compound, has been widely employed in various cell systems of the biopharmaceutical industry to reduce cell aggregation [35, 36]. Previous studies, including ours, have demonstrated that DS exhibited excellent performance in preventing excessive aggregation under hPSC suspension culture conditions without compromising cell viability and cellular pluripotency [23, 34, 37]. However, the signaling and molecular mechanisms underlying this phenomenon remain elusive. For hPSCs, cellular adhesion molecules (CAMs) play an important role in cell adhesion, attachment, and cell aggregation [38, 39], suggesting that the expression of CAMs may affect aggregation of hPSCs under suspension culture conditions. Therefore, we speculated that DS prevents hPSC aggregate adhesion through affecting the expression of CAMs. To test this hypothesis, we evaluated the expression of CAMs after DS treatment and determined the potential contribution of this signaling pathway.

## Materials and methods

### hPSC culture and maintenance

The hESC line, H9, was obtained from the WiCell Research Institute (Madison, WI, USA) under a Materials Transfer Agreement (No. 19-W0512) and was experimented during the 38th–52nd passages. The hiPSC line was provided as a gift by Dr. Liangxue Lai and was experimented during the 24th–29th passages. hPSC colonies were stably cultured on the hESC-qualified Matrigel (Corning, 354277)-supported adherent system, in mTeSR1 culture medium (STEMCELL Technologies, 85850), before being transferred to suspension culture. Cells were kept in a humidified incubator at 37 °C and 5% CO_2_, and the culture medium was refreshed daily. Routinely, cells were passaged as small clumps at a spilt ratio of 1:6 by ReLeSR (STEMCELL Technologies, 05872) every 5–6 days when they reached 80% confluence. Dextran sulfate (DS) compounds (Mw = 40,000) (Sigma‐Aldrich, 42867) were prepared by dilution in deionized water at a stock concentration of 100 mg/ml followed by 0.22-μm filter sterilization. Both H9 and hiPSC have been tested for experiments (Additional file 3: Fig. S1A-D).

### Aggregate suspension culture of hPSCs

To initiate suspension culture with aggregates, hPSC colonies cultured on hESC-qualified Matrigel-supported adherent systems were dissociated into single cells by Gentle Cell Dissociation Reagent (GCDR, STEMCELL Technologies, 07174), and then cells were counted by hemocytometer using trypan blue (Solarbio, C0040) staining and seeded into ultra-low attachment 6-well plates (Corning, 3471) at a cell density of 2 × 10^5^ cells/ml, and cultured in mTeSR1 medium with 10 μM Y-27632 (Selleck, S1049) in normoxic (21% O_2_) conditions for static suspension culture to form the aggregates. The medium was exchanged daily by angling the plates at 45° to allow aggregates to settle onto the bottom edge, and the DS compound was mixed with culture medium throughout the entire culture process at a final concentration of 100 μg/ml. Five days later, the morphology of hPSC aggregates was observed and photographed by phase contrast microscope. Cell counting evaluation was performed using a trypan blue staining after dissociation of aggregates into single cells by TrypLE (Thermo Fisher, 12604021). The diameter of aggregates was measured by ImageJ software.

### RNA extraction and quantitative reverse transcription polymerase chain reaction (qRT-PCR)

Total RNA was extracted using RNAiso Plus kit (Takara, 9109) according to the manufacturer’s manual. Following quantification in a NanoDrop microspectrophotometer (Thermo Fisher), 1 μg of RNA was used to synthesize cDNA using the PrimeScript™ RT Master Mix (Takara, RR036B). qRT-PCR was performed in triplicate using PowerUp™ SYBR™ Green (Thermo Fisher, A25742) on the Quant Studio™ 1 Real-Time PCR system (ABI, ABI7500). CT values for each sample were normalized against the corresponding expression of the housekeeping gene glyceraldehyde-3-phosphate dehydrogenase (GAPDH). The relative gene expression levels were quantified using the 2^−ΔΔ^CT method. The primer sequences for qRT-PCR used in the present study are listed in Additional file 1: Table S1.

### Flow cytometry (FC) analysis

hPSC colonies were dissociated into single cells by treatment with TrypLE, and cells were fluorescently labeled by incubation with PE antihuman OCT4 (OCT3) antibody (STEM CELL, 60093PE), PE antihuman TRA-1-81 antibody (STEM CELL, 60065PE), PE antihuman SSEA-4 antibody (STEMCELL, 60062PE), or PE mouse isotype-controlled antibody (BD, 556650). Fluorescence-positive cells were then detected using a BD FACS Celesta flow cytometer.

### Immunofluorescence (IF) staining

Aggregates were collected, washed with PBS, and fixed overnight in 4% paraformaldehyde at 4 °C, and then permeabilized with 0.5% Triton X‐100 for 20 min at 4 °C. Three washes with PBS were included between each step. Following washing, the samples were incubated in blocking buffer containing goat serum for 30–60 min at room temperature, then incubated in PBS containing primary antibodies such as rabbit anti-beta catenin antibody (Bioss, bs-23663R, 1:500) overnight at 4 ℃ followed by rewarming to room temperature and incubation in PBS containing secondary antibodies such as Alexa Fluor 594-conjugated goat anti-rabbit IgG (Cell Signaling Technology, 8889S, 1:800) for 1 h in the dark at room temperature. Nuclei were counterstained by incubation with DAPI (Solarbio, C0065) for 5 min, and the fluorescence signal was imaged on the single photon confocal microscopy (Ti-E A1, Nikon). The details of all antibodies used in the present study are listed in Additional file 2: Table S2.

### Western blot (WB)

Cells were lysed in RIPA lysis buffer (Solarbio, R0020) supplemented with PMSF (Solarbio, P0100) on ice. Protein concentrations were determined using a bicinchoninic acid (BCA) protein assay kit (Sangon Biotech, C503021) according to the manufacturer’s instructions. Western blots were performed in standard fashion. The primary antibodies used included rabbit anti-beta catenin antibody (Bioss, bs-23663R, 1:500), rabbit anti-MMP7 antibody (Bioss, bs-0423R, 1:500), rabbit anti-MMP-3 antibody (Bioss, bs-0413R, 1:500), rabbit anti-LEF-1 antibody (Bioss, bs-1843R, 1:500), rabbit anti-Frizzled 8 antibody (Bioss, bs-13219R, 1:500), rabbit anti-WNT7B antibody (Bioss, bs-6244R, 1:500), CD54/ICAM1 antibody (Cell Signaling Technology, 4915, 1:1,000), E-Cadherin Rabbit mAb (Cell Signaling Technology, 3915, 1:1,000), Slug Rabbit mAb (CST, 9585 T, 1:1,000), Twist-1 Antibody (R&D, MAB6230, 1:1,000), and anti-GAPDH antibody (Abcam, ab128915, 1:10,000). The secondary antibody was anti-rabbit IgG, HRP-linked antibody (Cell Signaling Technology, 7074, 1:3,000).

### Cell viability assay

The H9 cells were dissociated into single cells by GCDR, seeded at 5,000 cells per well in a 96-well plate coated with hESC-qualified Matrigel in the mTeSR1 medium supplemented with 10 μM Y-27632, and allowed to settle overnight. The next day, the medium was changed and supplemented with different concentrations of A-205804 (ICAM1 inhibitor, Selleck, S2885). The cell viability was measured using a Cell Counting Kit-8 (CCK-8, Beyotime, C0037). CCK-8 reagent was added to each well and incubated for 1 h, and the absorbance was read at 450 nm and recorded using a microplate spectrophotometer.

### Single-cell cloning assay

Ten thousand single cells of the H9 cells were inoculated on hESC-qualified Matrigel in a 6-well plate and cultured in mTeSR1 with 10 μM Y27632 on day 1; 100 μg/ml DS, 10 μM A-205804, DS plus 5 μM IWR-1-endo (Selleck, S7086), or 5 μM IWP2 (Selleck, S7085) were added in the experimental groups. Five days after seeding, cells were fixed with 4% paraformaldehyde for 20 min at room temperature, and colonies were visualized by alkaline phosphatase (AP) staining kit (Beyotime, C3206) according to the manufacturer’s instructions. The efficiency of cloning by single cell was assessed by counting the percentage of colony number versus cell number inoculated, and the area of colonies was calculated by ImageJ software. To count the cell number 24 h after inoculation, 1*10^5 single cells of H9 were inoculated on hESC-qualified Matrigel in a 12-well culture plate and cultured in mTeSR1 supplemented with 10 μM Y27632, 100 μg/ml DS, 10 μM A-205804, DS plus 5 μM IWR-1-endo, or 5 μM IWP2. Y27632 was removed by freshing the medium 12 h after seeding. The representative images of each group were observed and photographed by phase contrast microscope, and cell numbers were calculated by hemocytometer using trypan blue 24 h after culture.

### Integrated 3D hepatic differentiation of hPSC aggregates

The derivation of hepatic spheres from hPSC aggregates was performed as previously described [19]. In brief, after 5 days of culture, aggregates cultured with or without DS were collected, washed with PBS and then transferred to differentiation medium directly in an ultra-low 6-well plate. For the induction of endodermal cells, the basal medium consisted of RPMI 1640 (Thermo Fisher, 61870036), 1 × B27 (Gibco, 17504044) and 0.1% bovine serum albumin (BSA, Sigma, SRE0098), 6 μM CHIR99021 (Selleckchem, S1263) was added on day 1, and then 10 ng/ml Activin A (Peprotech, 120-14) was added for 2–3 days. For hepatic differentiation, aggregates were treated in DMEM/F12 (Gibco, 11330032) supplemented with 2% KSR, 10 ng/ml fibroblast growth factor 4 (FGF-4) (Peprotech, 100–31), and 10 ng/ml hepatocyte growth factor (HGF) (Peprotech, 100–39) for 6 days. Finally, for hepatocyte maturation, they were treated in the same medium plus 50% hepatocyte culture media without EGF (HCM, Lonza, CC-3198), 10 ng/ml oncostatin M (Peprotech, 300–10), and 10^–7^ M dexamethasone (Sigma, D4902) for another 12 days. On day 21, the hepatic spheres were collected and analyzed.

### ICAM1 knockdown

The doxycycline (Dox)-inducible short hairpin RNA (shRNA) targeting the human intercellular adhesion molecule 1 (ICAM1) gene and non-targeting scrambled shRNA (shNT), conjugated with GFP cloned in a lentivirus vector, were purchased from GeneCopoeia. The target sequence of shNT was GCTTCGCGCCGTAGTCTTA, the target sequences of shRNAs included four groups designated as a, b, c, and d, and their sequences were GCTGACGTGTGCAGTAATACT, GCCAGCTTATACACAAGAACC, CCAACCAATGTGCTATTCAAA, and GGTATGAGATTGTCATCATCA, respectively. To produce lentivirus, 5 μg of plasmid was transfected into the 293T cell line (cells are 70%–80% confluent at the moment of transfection), together with 3.75 μg of PSPAX2 and 1.25 μg PMD2.G by Lipofectamine 3000 Transfection Reagent (Thermo Fisher, L3000015). Virus-containing supernatant was harvested at 48 and 96 h after transfection. Following centrifugation to remove cell debris, supernatant was filtered through 0.45-μm filters, and then ultracentrifugation was performed at 25,000 g, 4 ℃ for 2 h. Finally, the small pellet of lentivirus was dissolved with PBS on ice and spilt into centrifuge tubes, aliquoted, and kept at − 80 °C until use. The titer of virus stock was determined by Lenti-X p24 Rapid Titer Kit (Takara, 632200). To transduce lentivirus particles into H9 cells, 10^5^ single H9 cells were seeded into hESC-qualified Matrigel-coated 6-well culture plates. The next day, 990 μl of fresh medium supplemented with 10 μl of virus stock and 2 μg/ml polybrene (Sigma, TR-1003) was changed for each well. Then, the medium was replaced with fresh medium after 12 h of incubation. When passaged, 0.5 μg/ml puromycin was added for the selection of transduced cells, and then GFP positive colonies were picked up to passage for obtaining the stable transduced H9 cells. Afterward, doxycycline (Dox) was added to the medium at a final concentration of 1 μg/ml for 48 h to select the most efficient shRNA for further knockdown experiments. 1 μg/ml Dox was added into the medium to induce the expression of shNT; four shRNA were screened through the efficiency of knockdown of ICAM1 by qRT-PCR. The fluorescence images were observed and photographed by JuLI Stage Real-Time Cell History Recorder (NanoEntek, JS1000S).

### Subcellular fractionation

The H9 aggregates with different treatments were collected, washed with PBS, then harvested in 350 μl of hypotonic buffer (10 mM Hepes, pH 7.4, 42 mM KCl, 5 mM MgCl_2_), and incubated for 30 min on ice. The cells were lysed by passing through a 25-gauge needle 10 times. The cytosolic fraction (supernatant) was collected by centrifugation at 200 g for 10 min at 4 °C. The nuclear pellet was washed twice by resuspending in 500 ml of hypotonic buffer and passed through a 25-gauge needle10 times. The nuclei were pelleted by centrifugation at 3000 g for 10 min at 4 °C. Isolated cytosolic and nuclear fractions were resuspended in lysis buffer, respectively. The loading buffer was added, followed by boiling for 5 min, and then subjected to immunoblot analysis.

### Statistical analysis

Data are expressed as the mean ± standard deviation (n = 3). Statistical analysis was performed using GraphPad Prism 6; the unpaired Student’s t test and one-way ANOVA (Tukey correction for multiple comparisons) were used to evaluate statistical significance. Differences were considered statistically significant at *P* < 0.05.

## Results

### DS treatment prevents excessive aggregation and promotes hepatic differentiation of the H9 cells in 3D suspension culture

**Fig. 1:**
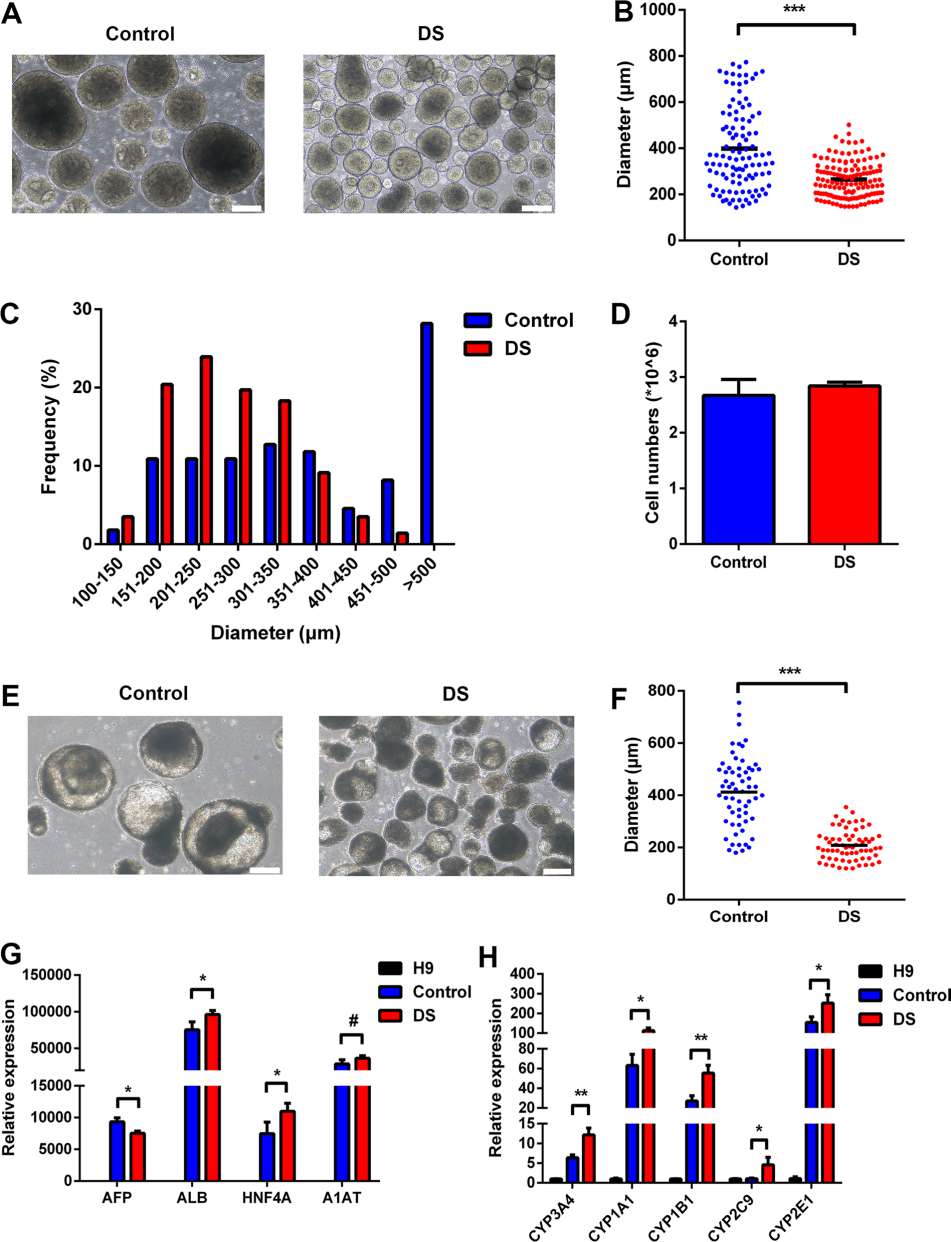
The effect of DS on H9 aggregate sizes and further integrated hepatic differentiation in 3D suspension culture conditions. **A** Representative images of the H9 aggregates on day 5 after treatment with 100 μg/ml DS. Scale bar = 200 μm. **B** Comparison of average diameter of the H9 aggregates on day 5. **C** Diameter distribution of the H9 aggregates treated with or without DS on day 5. **D** Comparison of the cell numbers after 5 days of culture. **E** Representative images of hepatic spheres on day 15 after hepatic differentiation induction, the morphology of hepatic spheres in the control group was big and hollow, and it was relatively small and dense in DS group. Scale bar = 200 μm. **F** Comparison of average diameter of the hepatic aggregates from control and DS group at day 21 of hepatic differentiation. **G** Gene expression analysis by qRT-PCR for hepatic marker genes AFP, ALB, HNF4A, and A1AT from different treatment on the H9 aggregates. (H) Gene expression analysis by qRT-PCR for CYP450 including CYP3A4, CYP1A1, CYP1B1, CYP2C9, and CYP2E1. Relative gene expression represents data normalized to GADPH and expressed relative to undifferentiated H9. Data represent the mean ± SD. *P < 0.05, **P < 0.01, and ***P < 0.001. Abbreviations: AFP, α-fetoprotein; ALB, albumin; HNF4A, hepatocyte nuclear factor 4 alpha; A1AT, alpha-1 antitrypsin; CYP, cytochrome P450 family

In line with previous studies, including ours [23, 34], the addition of 100 μg/ml DS significantly decreased the diameter of H9 aggregates on day 5 in 3D static suspension culture conditions (Fig. 1A–C). More small-sized and homogeneous aggregates with reduced standard deviations were observed in the presence of DS. The average diameter of aggregates reached 403 ± 182 μm in the absence of DS, which was 50% bigger than that of aggregates in DS group (Fig. 1B). Less than 5% of the aggregates had an average diameter more than 400 μm after DS treatment, as opposed to 30% in the control group (Fig. 1C). In addition, DS treatment did not reduce the proliferation capacity of H9 aggregates (Fig. 1D). In addition, these results were repeated with hiPSCs and obtained similar tendency (Additional file 4: Fig. S2A-D). To estimate the effect of DS on further 3D differentiation of H9 aggregates, we collected the aggregates with or without DS treatment after 5 days of static suspension culture and transferred them into hepatic differentiation medium [19]. After 15 days of differentiation, the hepatic spheres were collected for analysis. Similar to the situation in mTeSR1, DS-treated small-sized aggregates differentiated into smaller and relatively dense hepatic spheres (208 ± 57 μm). Without DS treatment, bigger aggregates became cystic and bigger-sized hepatic spheres (412 ± 133 μm) after the differentiation process (Fig. 1E, F). Furthermore, the hepatic spheres in the DS-treated group expressed greater levels of mature hepatocyte markers like albumin (ALB), HNF4A and α1-antitrypsin (A1AT), and lower levels of the immature hepatocyte marker, alpha fetoprotein (AFP) (Fig. 1G), indicating that DS treatment promoted the hepatic differentiation of hPSC aggregates in suspension culture due to a relative smaller size of aggregates. Moreover, the expression of several important phase I drug-metabolizing enzymes, including CYP3A4, CYP1A1, CYP1B1, CYP2C9, and CYP2E1, were also significantly higher than those in the control group (Fig. 1H). These results demonstrated that DS treatment not only enabled the formation of uniform and small-sized aggregates, but it also was beneficial for 3D hepatocyte differentiation [23].

### Distinct CAMs expression after DS treatment

**Fig. 2:**
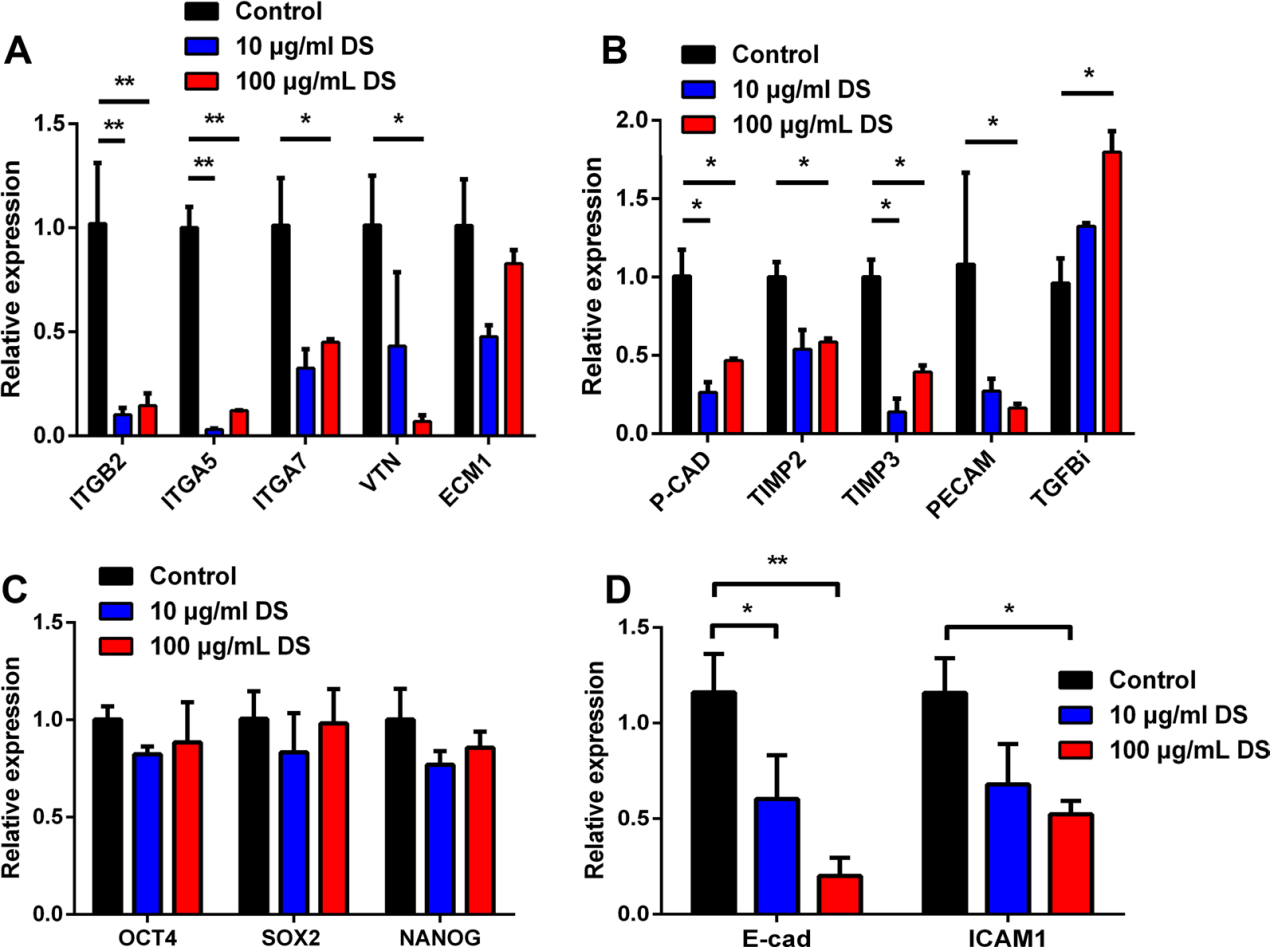
The effect of DS on the expression of CAMs on the H9 aggregates in 3D suspension culture conditions. **A**–**D** Gene expression analysis by qRT-PCR for CAMs-related genes (**A**), cell adhesion-associated genes, inhibitors of the matrix metalloproteinases, and TGFβi (**B**), pluripotent genes (**C**), and E-cad and ICAM1 (**D**) after treatment with various DS concentrations. Relative gene expression represents data normalized to GADPH and expressed relative to untreated H9 aggregates. Data represent the mean ± SD. *P < 0.05 and **P < 0.01. Abbreviations: OCT4, POU class 5 homeobox 1; NANOG, Nanog homeobox; SOX2, SRY-box transcription factor 2; P-cad, cadherin 3; TIMP, TIMP metallopeptidase inhibitor; ITGA, integrin subunit alpha; TGFβi, transforming growth factor beta induced; VTN, vitronectin; ECM1, extracellular matrix protein 1; PECAM, platelet and endothelial cell adhesion molecule 1; E-cad, cadherin 1; ICAM1, intercellular adhesion molecule 1

Previous studies suggested that, for hPSCs, the differently adhesive ability of aggregates in suspension culture correlated with the expression of CAMs [40]. Therefore, we speculated that the prevention of excessive aggregation by DS might be caused by differentially expressed CAMs. To verify this hypothesis, we first compared the expressions of a panel of adhesion-related genes and pluripotency-related genes between DS groups and control groups by qRT-PCR. Notably, genes related to extracellular matrix (e.g., vitronectin (VTN) and extracellular matrix protein 1 (ECM1)), a variety of integrin molecules (e.g., integrin subunit alpha 5 (ITGA5), integrin subunit alpha 7 (ITGA7) and integrin subunit beta 2 (ITGB2)), genes directly associated with cell adhesion (e.g., cadherin 3 (P-cad) and platelet/endothelial cell adhesion molecule 1 (PECAM)), and natural inhibitors of the matrix metalloproteinases (e.g., tissue inhibitors metalloproteinases2/3 (TIMP2/3)) were down-regulated after treatment with DS (Fig. 2A, B). In addition, the expression of transforming growth factor beta induced (TGFβi) was up-regulated after DS treatment, which would result in inhibiting cell adhesion (Fig. 2B) [41]. However, the expressions of pluripotent genes OCT4, SOX2, and NANOG did not exhibit significant differences (Fig. 2C). In terms of genes related to CAMs, the expression of E-cadherin (E-cad) and ICAM1 which are highly expressed in H9 cells was also significantly down-regulated after DS treatment (Fig. 2D). E-cad is highly correlated with hPSC adhesion and attachment [38, 42]. For various tumor cell lines, ICAM1 plays an important role in cell aggregation under suspension cultures [39, 43]; however, to the best of our knowledge, there are no reports about the roles of ICAM1 in hPSC adhesion. Therefore, we focused on the roles of E-cad and ICAM1 to determine whether DS functioned through these two CAMs.

### Distinct expressions of ICAM1 between big and small aggregates

**Fig. 3:**
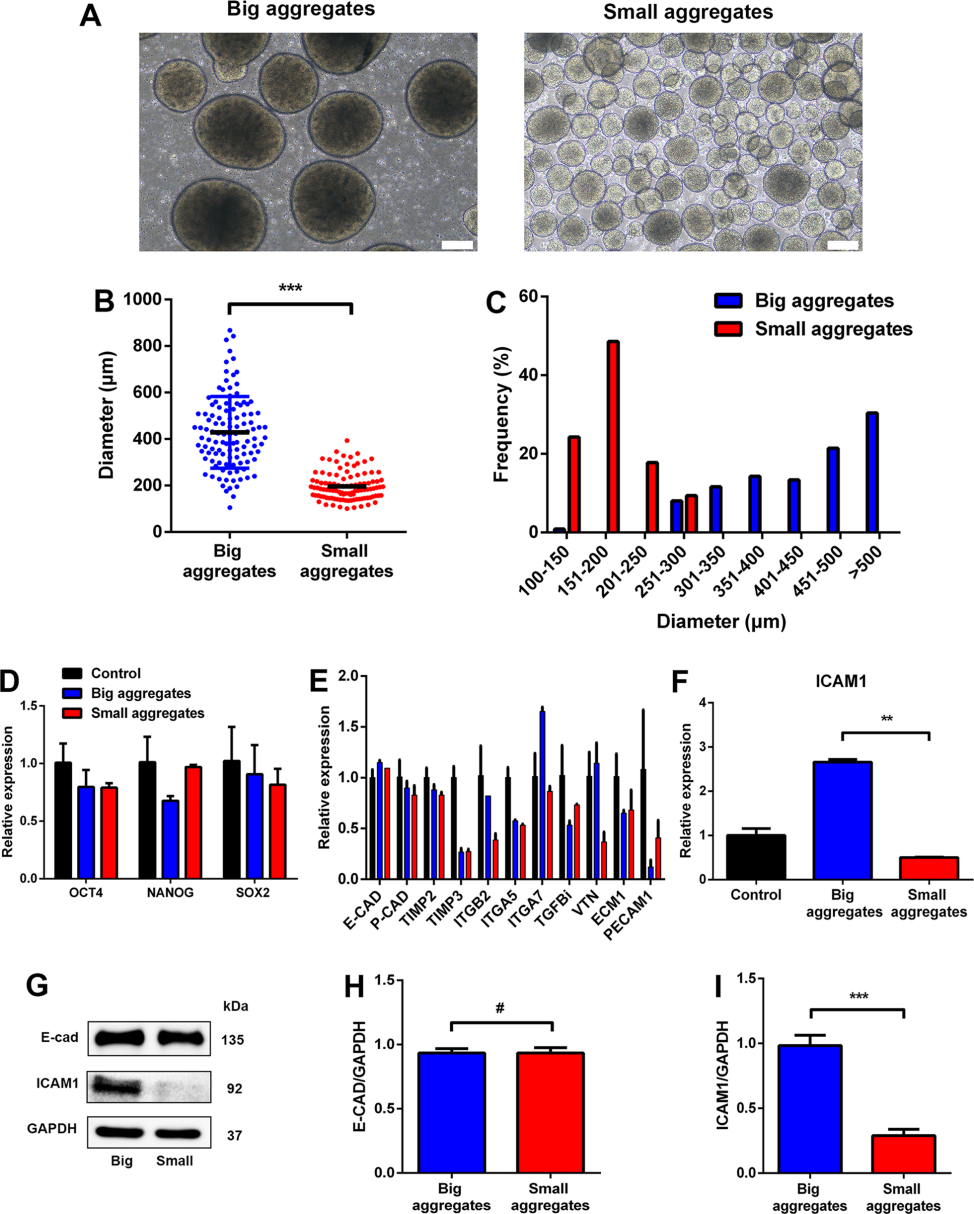
The expression of ICAM1 was significantly different between naturally developed big and small aggregates of the H9 cells. **A** Representative images of big and small aggregates of the H9 cells on day 5 in 3D suspension culture condition without any extra treatment; these two groups were separated by natural settlement. Scale bar = 200 μm. **B** Comparison of average diameter of big and small aggregates of the H9 cells on day 5. **C** Diameter distribution of big and small aggregates of the H9 cells on day 5. **D**–**F** Gene expression analysis by qRT-PCR for pluripotent genes (**D**), CAMs-related genes (**E**), and ICAM1 (**F**), relative gene expression represents data normalized to GADPH and expressed relative to untreated H9 aggregates. **G** The protein levels of E-cad and ICAM1 were determined by western blotting in big and small aggregates of the H9 cells. **H**, **I** The densitometry for the protein levels of E-cad (**H**) and ICAM1 (**I**) were quantitated in G; GAPDH was used as a loading control. Data represent the mean ± SD. *P < 0.05 and **P < 0.01 and ***P < 0.001

By using repeated natural sedimentation, we separated H9 aggregates which were expanded under static suspension culture conditions for 5 days into big and small aggregates, respectively (Fig. 3A). The average diameter of big aggregate group was 400 ± 153 μm which is nearly twofold of that of small aggregate group (Fig. 3B), and more than 60% of the aggregates were bigger than 400 μm in the diameter. Approximately 90% of the aggregates were smaller than 250 μm in diameter in the small aggregates group (Fig. 3C). However, qRT-PCR results revealed that there was no obvious difference on the expression of pluripotent genes and E-cad between the two groups (Fig. 3D, E). Notably, among the CAMs detected, only ICAM1 was expressed differentially, which was fivefold higher in the big aggregate group than in the small one (Fig. 3F). The difference of ICAM1 between two groups was further confirmed by protein expression through western blot analysis, while the protein level of E-cad remained comparable (Fig. 3G–I). These results indicated that ICAM1 might play an important role in affecting the size of aggregates or adhesion ability of aggregates in 3D suspension cultures.

### Modulation of the size of aggregates by regulating the expression of ICAM1

**Fig. 4:**
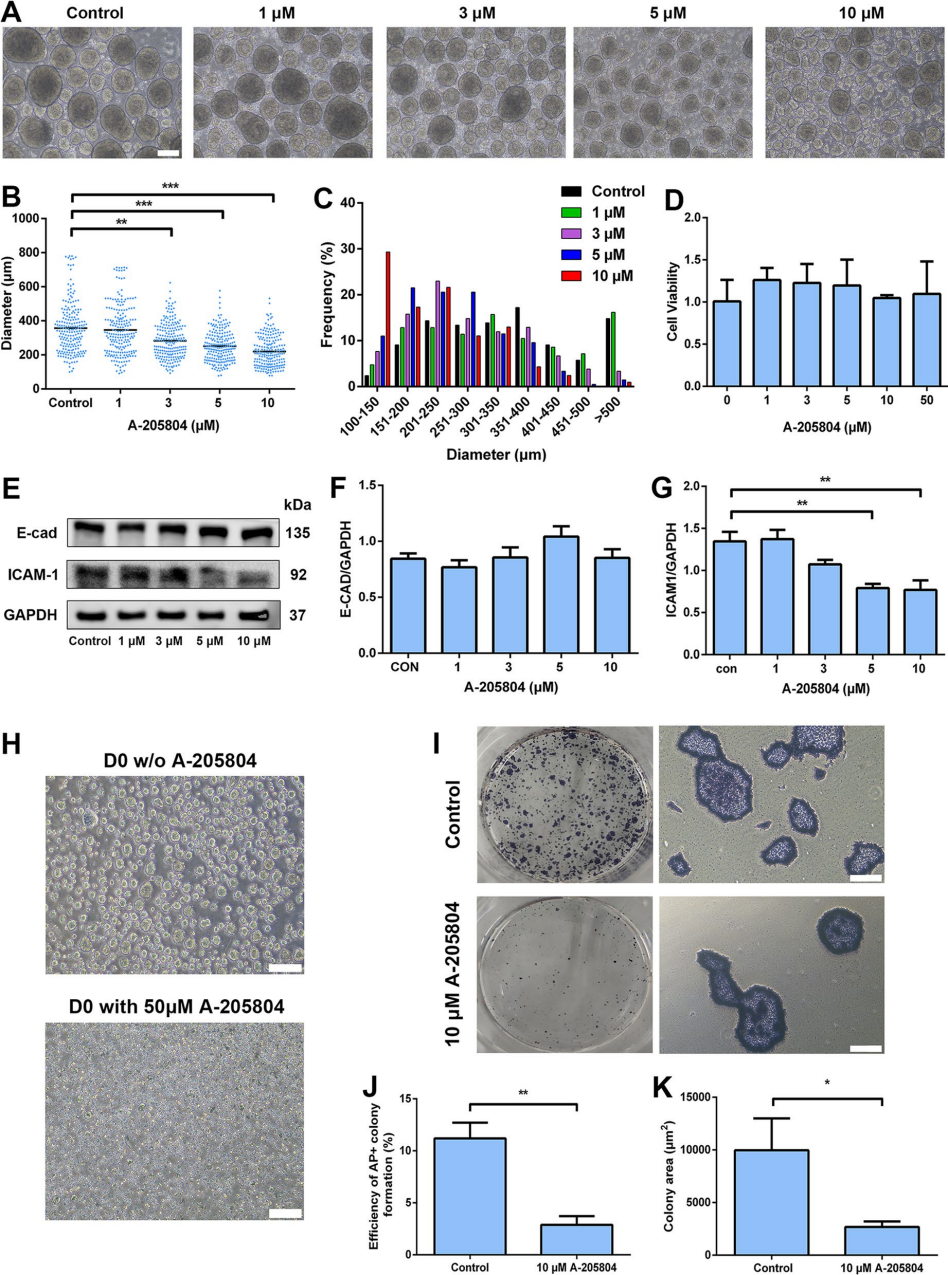
Controlling the size of the aggregates of the H9 cells by ICAM1 inhibitor A-205804. **A** Representative images of the aggregates of the H9 cells after treatment with various concentrations of A-205804 on day 5. Scale bar = 200 μm. **B** Comparison of average diameter of the aggregates of the H9 cells after treatment with various concentrations of A-205804 on day 5. **C** Diameter distribution of the aggregates of the H9 cells after treatment with various concentrations of A-205804 on day 5. **D** Cell viability of the H9 cells assayed by CCK-8 after treatment with various concentrations of A-205804. **E** The protein levels of E-cad and ICAM1 were determined by western blotting in aggregates of the H9 cells after treatment with various concentrations of A-205804. **F**, **G** The densitometry for the protein levels of E-cad **F** and ICAM1 **G** were quantitated in E; GAPDH was used as a loading control. **H** Representative images show the addition of 50 μM A-205804 to the inoculum abolished aggregate formation completely, Scale bar = 200 μm. **I** AP staining of the H9 colonies with or without adding A-205804; single cells were seeded in six-well culture plates coated with Matrigel, 10 μM Y-27632 was added on day 1 in both groups. Representative images are shown. Scale bar = 200 μm. **J** Quantification of the efficiency of AP positive colonies in I. (K) Quantification of the average colony area in I. Data represent the mean ± SD. *P < 0.05 and **P < 0.01 and ***P < 0.001

To assess whether regulation of ICAM1 expression could modulate the size of aggregates in 3D suspension cultures, we utilized the ICAM1 specific inhibitor, A-205804 to test this hypothesis. After treatment with various concentrations of A-205804 under 3D suspension culture condition, we found that the size of aggregates exhibited a tendency to decrease in a dose-dependent manner (Fig. 4A–C). 67% of the aggregates were smaller than 250 μm in diameter, and only 3% of the aggregates were bigger than 400 μm in diameter in the group treated with 10 μM A-205804, while only 30% of the aggregates were smaller than 250 μm in diameter, and 25% of the aggregates were bigger than 400 μm in diameter in the control group (Fig. 4C). Importantly, no obvious difference in cell viability was observed regardless of A-205804 concentrations investigated (Fig. 4D), indicating that the decrease in the aggregate size was not the results of toxicity caused by the higher dose of the inhibitors. As expected, the expression level of ICAM1 protein also was decreased with the increased dose of A-205804 (Fig. 4E–G), but E-cad levels remained unchanged (Fig. 4E, F), consistent with aforementioned results. In addition, by adding 50 μM A-205804 into medium at the time point of seeding, we found that high-dosed ICAM1 inhibitor would impede the aggregation of the H9 cells, whereas formation of aggregates was observed in the control group as expected (Fig. 4H). This concentration of A-205804 did not affect cell viability (Fig. 4D), indicating that the expression of ICAM1 is important for aggregate formation, and it appears that the use of the inhibitor was an effective approach to decrease the size of aggregates through interfering with ICAM1 expression in 3D suspension cultures. To further investigate whether ICAM1 has an effect on extracellular adhesion during the proliferation of hPSCs, we performed a single-cell cloning assay with or without treatment with 10 μM A-205804. After 5 days of culture, the formation efficiency of the alkaline phosphatase (AP)-positive colonies and the size of the average colony area in the control group were significantly higher than in the conditioned group (Fig. 4I–K), demonstrating that the expression of ICAM1 was crucial to extracellular adhesion even in 2D adhesion culture conditions.

**Fig. 5:**
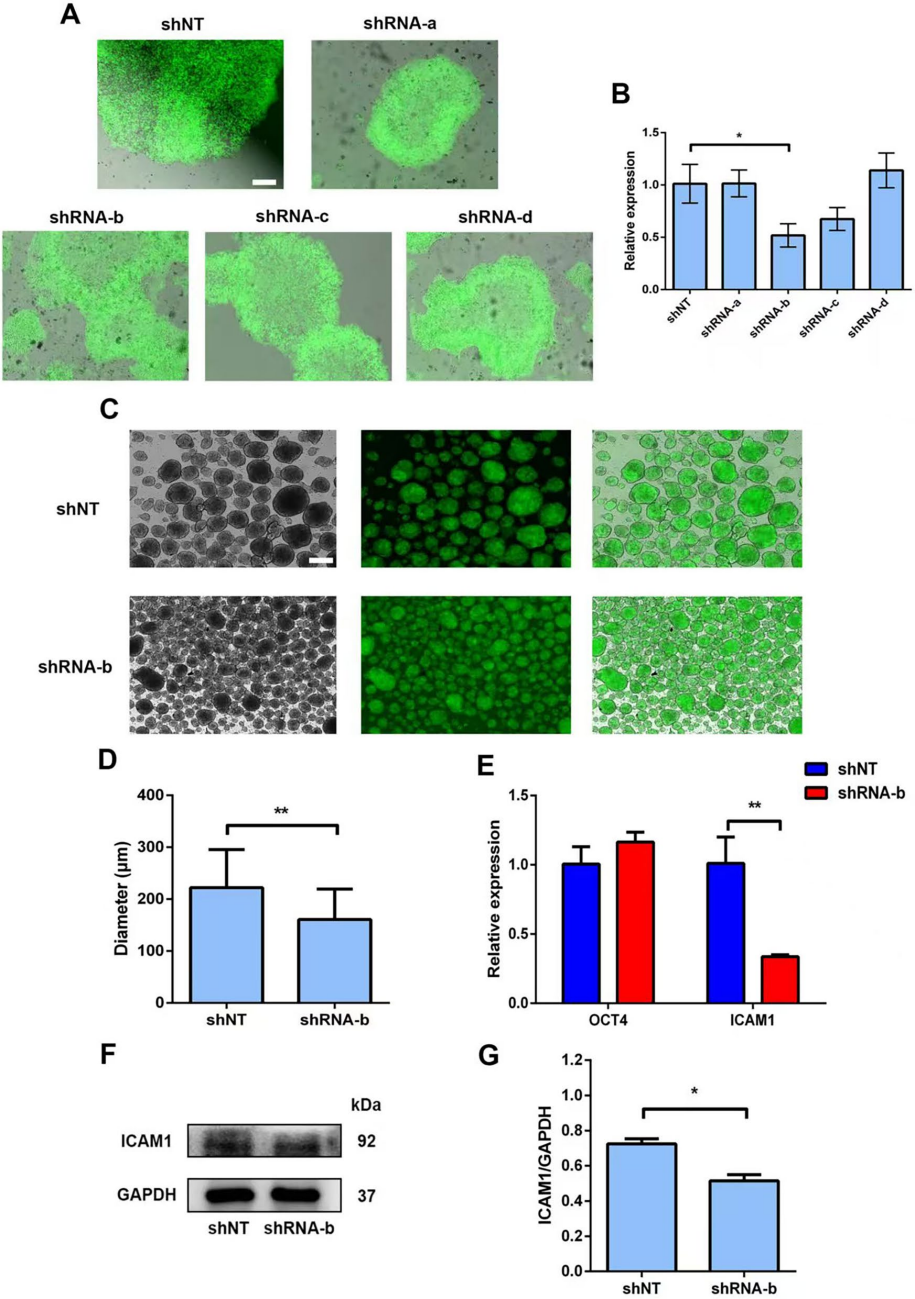
Interfering ICAM1 endogenously by lentivirus-mediated shRNA. **A** The EGFP^+^ colonies after lentivirus transduction and puromycin selection. Representative images were shown. Scale bar = 200 μm. **B** Gene expression analysis by qRT-PCR for ICAM1 in the H9 cells transduced with the lentivirus expressing different shRNAs, relative gene expression represents data normalized to GADPH and expressed relative to cells transduced with the lentivirus expressing shNT. **C** The EGFP^+^ aggregates were shown in suspension culture on day 5. Representative images were shown. Scale bar = 200 μm. **D** Comparison of average diameter of the aggregates of the H9 cells in two groups. **E** Gene expression analysis by qRT-PCR for ICAM1 and OCT4 in the aggregates transduced with the lentivirus expressing shRNA-b and shNT, respectively, relative gene expression represents data normalized to GADPH and expressed relative to cells transfected by shNT. (F) The protein levels of E-cad and ICAM1 were determined by western blotting in the aggregates transduced with the lentivirus expressing shRNA-b and shNT, respectively, in two groups. **G** The densitometry for the protein levels of ICAM1 was quantitated in F; GAPDH was used as a loading control. Data represent the mean ± SD. *P < 0.05 and **P < 0.01

Next, knockdown of endogenous ICAM1 by shRNA was performed to further determine the function of ICAM1 expression on extracellular adhesion. A homogenous population of EGFP-positive colonies was observed (Fig. 5A), indicating a high transduction rate of a lentiviral vector co-expressing shNT/shRNAs and EGFP in the H9 cells. Through screening the knockdown efficiency of ICAM1-shRNAs (Fig. 5A, B), shRNA-b which significantly down-regulated ICAM1 was selected for further 3D suspension culture experiments. Dox was added to both groups, shNT and shRNA-b, to a final concentration of 1 µg/ml to induce the expression of shRNA. On day 5 after culture, cells transduced with shRNA-b exhibited significant smaller-sized aggregates when compared to those in the shNT group (Fig. 5C, D), further demonstrating that ICAM1 was an important factor in the induction of cellular adhesion. Although the expression of ICAM1 was significantly down-regulated, the expression of OCT4, one of the most important pluripotent genes, remained unchanged (Fig. 5E), indicating that ICAM1-shRNA only interfered with the expression of ICAM1 rather than affecting the pluripotency of the H9 cells. The expression of ICAM1 protein was further confirmed by western blot analysis (Fig. 5F, G). These results together showed the importance of the expression of ICAM in hPSCs for cell adhesion and for controlling aggregate sizes in 3D suspension culture.

### Disturbance of aggregate adhesion by DS-mediated E-CAD reduction acted through the activation of the canonical Wnt signaling pathway

**Fig. 6:**
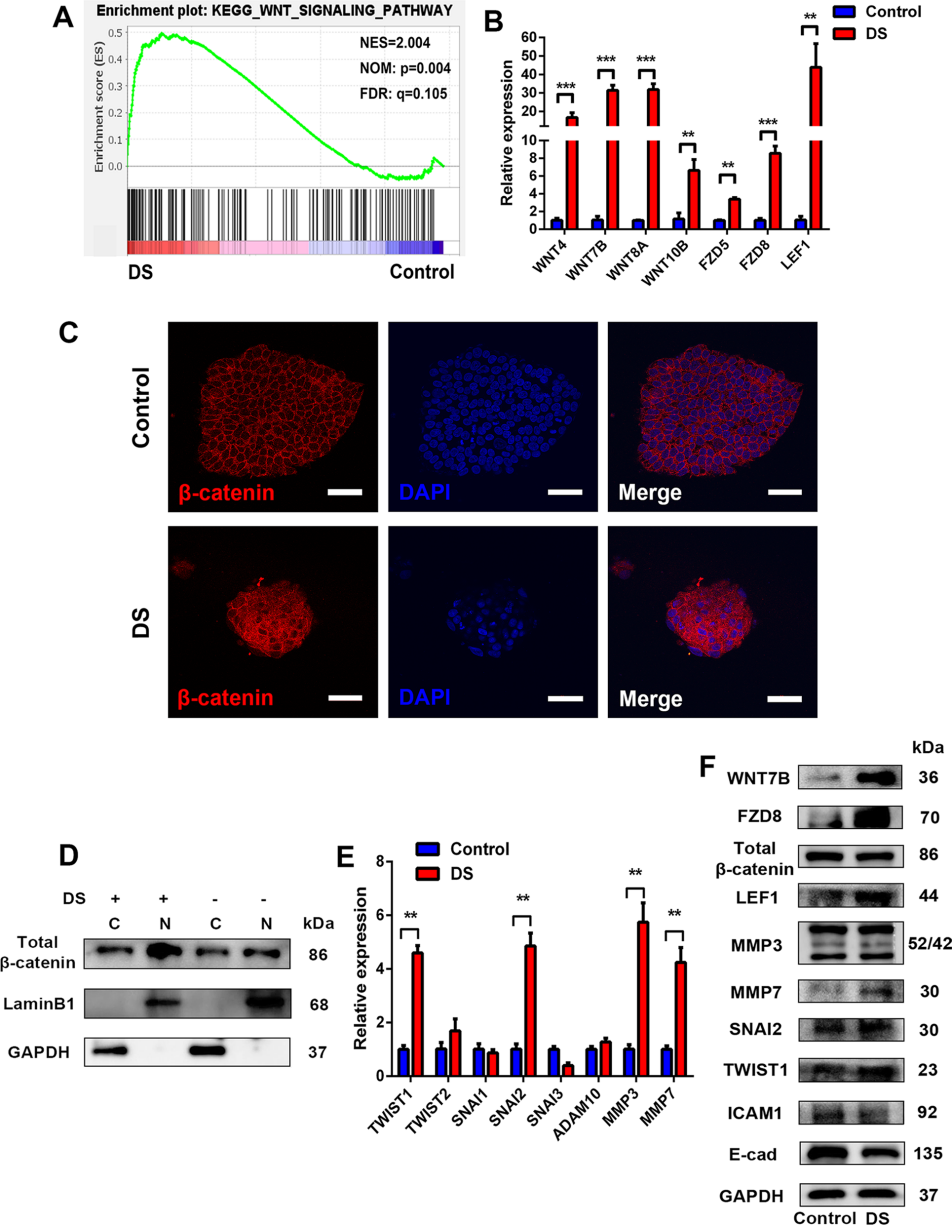
DS regulated the adhesion of the aggregates of the H9 cells via canonical Wnt signaling. **A** GSEA analyses of Wnt-associated genes enriched in the aggregates of the H9 cells treated with DS. **B** Gene expression analysis by qRT-PCR for Wnt-associated genes, relative gene expression represents data normalized to GADPH and expressed relative to untreated aggregates. **C** Immunofluorescence analysis of β-catenin (red) on the aggregates of the H9 cells treated with or without DS; the nuclei were stained with DAPI. Scale bars = 50 μm. **D** The protein levels of total β-catenin in nuclei and cytoplasm were determined by western blotting in the aggregates of the H9 cells after treatment with or without DS; the purity of nuclear and cytosolic fractions was verified using laminB1 and GAPDH, respectively. **E** Gene expression analysis by qRT-PCR for Wnt-targeted genes; relative gene expression represents data normalized to GADPH and expressed relative to untreated aggregates. **F** The protein levels of WNT7B, FZD8, total β-catenin, LEF1, MMP3, MMP7, TWIST1, SNAI2, and E-cad were determined by western blotting in the aggregates of the H9 cells after treatment with or without DS; GAPDH was used as a loading control. Data represent the mean ± SD. *P < 0.05 and **P < 0.01 and ***P < 0.001. FZD, frizzled class receptor; LEF1, lymphoid enhancer-binding factor 1; TWIST, twist family bHLH transcription factor; SNAI, snail family transcriptional repressor; MMP, matrix metallopeptidase

Previous studies have revealed that in 3D suspension cultures of hPSCs, the secreted Wnt antagonists were significantly up-regulated and led to the down-regulation of canonical Wnt-targeted genes when compared with 2D adherent cultures, and they further enhanced the expression of the central adherens junction components, such as E-cad [44]. In our previous study, we performed RNA-seq analyses to investigate the mechanisms by which DS treatment controlled the size of the H9 cell aggregates [23]. In the present study, as indicated by KEGG analysis, we determined that the Wnt signaling pathway was significantly up-regulated after DS treatment (Fig. 6A). These results were further verified by qRT-PCR and western blot analysis. Several Wnt ligands (WNT4, WNT7B, WNT8A, and WNT10B), receptors (FZD5 and FZD8), and a gene involved in the Wnt signaling pathway (LEF1) were highly expressed, associated with the decrease in E-CAD after DS treatment (Fig. 6B). Furthermore, with the up-regulation of Wnt signaling, we observed a higher translocation of β-catenin into the nucleus by immunofluorescence staining and western blot analysis (Fig. 6C, D), indicating the activation of the canonical Wnt signaling pathway [45]. In addition, there are several transcription factors (TFs) which are Wnt target genes down-regulating the expression of E-cad, including the twist family bHLH transcription factor (Twist), snail family transcriptional repressor (Snai), and the matrix metallopeptidase (MMP) families [46, 47]. We detected the gene expression patterns of these three families by qRT-PCR and found that the expressions of Twist1, Snai2, MMP3, and MMP7 were significantly up-regulated after DS treatment in the H9 aggregates under 3D suspension culture conditions (Fig. 6E), and the protein expression levels of these TFs were further confirmed by western blot analysis, which was consistent with the qRT-PCR analysis (Fig. 6B, E, F). These results indicated that DS treatment could suppress the expression of E-cad through activating the canonical Wnt signaling pathway.

**Fig. 7:**
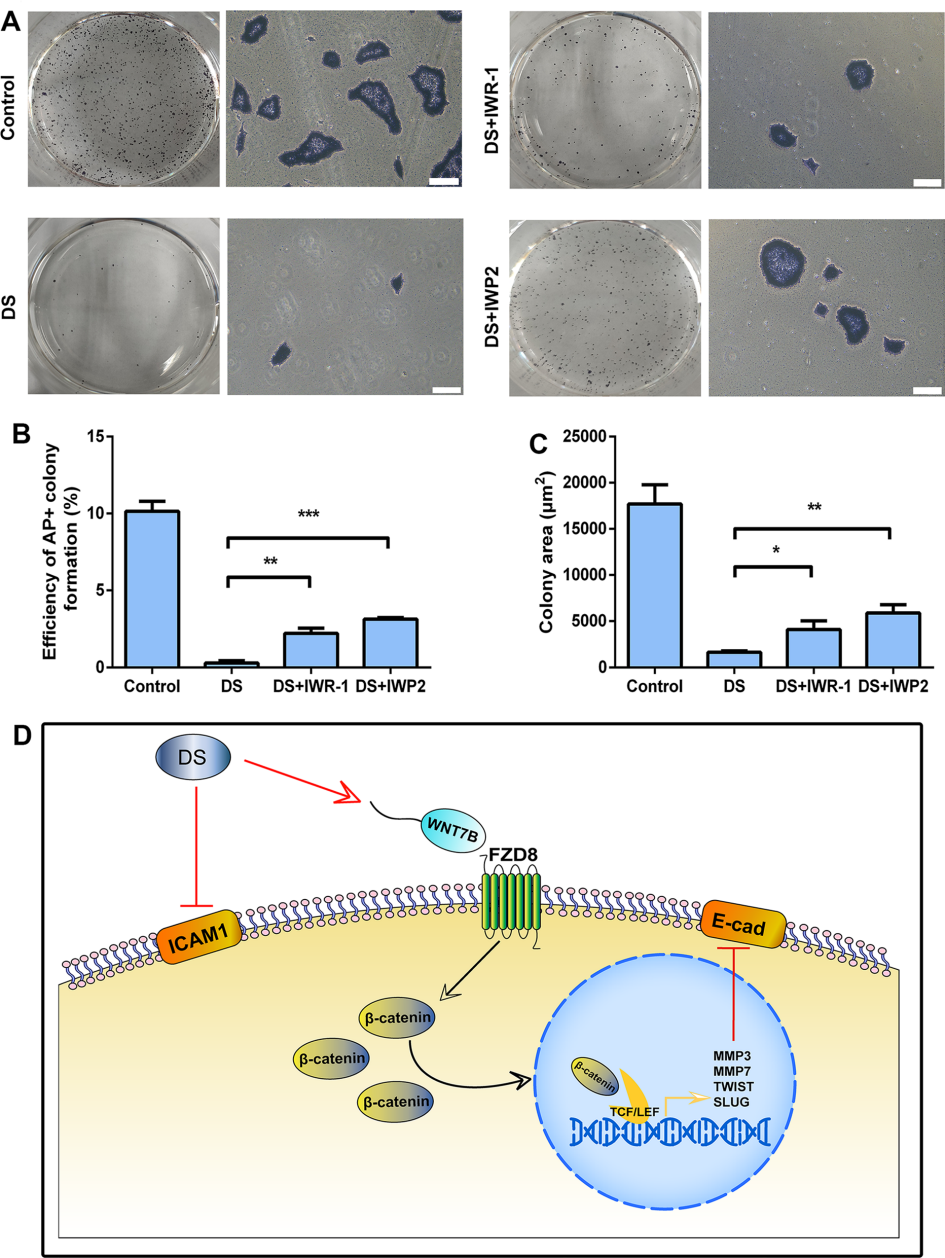
The effect of DS on adhesion of the H9 cells could be partially rescued by Wnt inhibitor. **A** AP staining of the H9 colonies treated with or without DS, supplemented with 5 μM Wnt inhibitor IWR-1 or IWP2, single cells were seeded in six-well culture plates coated with Matrigel, 10 μM Y-27632 was added on day 1 in all groups. Representative images were shown. Scale bar = 200 μm. **B**, **C** Quantification of the efficiency of AP^+^ colonies (**B**) and the average colony area (**C**) in A. **D** Schematic diagram showing DS prevents the adhesion of the aggregates of the H9 cells through modulating the expression of ICAM1 and the canonical Wnt signaling pathway. Treating the aggregates with DS leads to the down-regulation of the expression of ICAM1. The expression of secreted Wnt ligands and receptor such as Wnt7B and FZD8 is up-regulated in 3D suspension culture conditions after DS treatment, causing the activation of the canonical Wnt signaling pathway, which impedes the degradation of cytosolic β-catenin and promotes its transference into the nucleus, thus further up-regulating the expression of Wnt target genes, consequently down-regulating the transference expression of E-cad. Data represent the mean ± SD. *P < 0.05 and **P < 0.01 and ***P < 0.001

The expression of E-cad in hPSCs is indispensable for cell adhesion and aggregation under 3D culture conditions [38, 42]. Thus, we further performed single-cell colony assay to confirm whether DS treatment could disturb the adhesion of hPSCs and to verify whether Wnt signaling inhibitors could rescue this phenomenon under 2D culture conditions. As expected, the formation efficiency of the single-cell colony and the average area of colonies were decreased after DS treatment (Fig. 7A–C). However, the colony-forming efficiency and the average area of colonies could be rescued partially by adding Wnt inhibitors, 5 μM IWR-1 and 5 μM IWP2, respectively (Fig. 7A–C). In order to decouple the effect of proliferation on colony formation assay, we also counted the cell number 24 h after inoculation. As expected, the cell number was decreased prominently after DS treatment and could be rescued by Wnt inhibitors (Additional file 5: Fig. S3A-B). Moreover, we also conducted rescue assay with Wnt inhibitors under 3D suspension culture condition. By adding 5 μM IWR-1 or 5 μM IWP2 with DS into mTeSR1, the average size of aggregates was notably bigger than those treated with DS only, but still smaller than aggregates in control group (Additional file 6: Fig. S4A-C) after 5 days of culture. Interestingly, the expression of E-cad was also up-regulated after the treatment with Wnt inhibitors (Additional file 6: Fig. S4E). In addition, there is still no obvious difference on the cell number between each group (Additional file 6: Fig. S4D). These results further demonstrated that DS functioned through the Wnt signaling pathway.

In order to determine whether the functions of DS on hPSC aggregates could be replaced by ICAM1 inhibitor and Wnt agonist directly, further investigation was performed. To address this issue, we replaced DS with 10 μM A-205804 plus 3 μM CHIR99021 or 10 ng/ml Wnt3A (both are Wnt agonist) and cultured H9 aggregates for 5 days. After the treatment with ICAM1 inhibitor and WNT agonist, the sizes of aggregates were not uniform and exhibited heterogeneity (Additional file 7: Fig. S5A) when compared to those in control; moreover, these H9 aggregates no longer maintained the stable expression of three core pluripotent genes, OCT4, SOX2, and NANOG (Additional file 7: Fig. S5B). Surprisingly, 5 days after the aforementioned treatment, H9 aggregates spontaneously differentiated into three germ layers (mesoderm, endoderm, and ectoderm) determined by qRT-PCR analysis (Additional file 7: Fig. S5C). It appears that the effect of DS could not be replaced by WNT agonist. WNT signaling pathway is a more complicated system and plays a large number of functions in cell; DS treatment most likely induced a specific pathway indicated in our study; however, WNT agonist might induce more pathways which might inhibit the pluripotency and trigger the differentiation of hPSC. Thus, the mechanism of DS treatment on hPSCs still needs future work to investigate thoroughly.

Taken together, our results revealed that DS treatment interfered with the expression of E-cad through activation of the canonical Wnt signaling pathway and further co-modulated the adhesion of hPSC aggregates with DS-mediated ICAM1 reduction in 3D suspension culture conditions (Fig. 7D).

## Discussion

The unlimited self-renewal ability and multi-lineage differentiation potential of hPSCs emphasize their potential use in clinical treatment and in the pharmaceutical industry [48, 49]. In order to take full advantage of hPSCs, large-scale expansion is indispensable in order to obtain enough cells for further applications. The bottleneck for large-scale production of clinical quantities of hPSCs currently is the lack of an appropriate scalable bioprocess protocol [50].

By utilizing stirred-tank bioreactors, several laboratories have established expandable protocols for hPSC aggregates using 3D suspension culture systems [10, 11, 17, 25, 26, 51]; the best of these protocols obtained 70-fold cell expansion in 7 days and achieved a density of 3.5 × 10^7^ cells/ml [26]. However, the major limitation still unsolved was the complex hydrodynamics and high shear stress at the tip of impeller, which could be injurious to stem cell viability and differentiation [29–31]. Furthermore, it was not as easy as reported to control aggregate sizes by different agitation rates in stirred-tank bioreactors [17]. Borys and colleagues demonstrated that they failed to generate consistent hiPSC aggregates in all tested agitation rates in horizontal-blade stirred-tank bioreactors, most likely because aggregates were limited from moving throughout the entire volume of the bioreactor [17]. Actually, controlling hPSC aggregate sizes in 3D suspension culture systems is the most difficult and critical process. To date, there has not been a good solution for preventing hPSC excess adhesion and aggregation formation, thus limiting the production of clinical quantities of hPSCs. In general, properly combining the physical approaches with biochemical approaches may be the best solution to control aggregate sizes of hPSCs. For example, by stirring the impellers at a low agitation rate, it not only avoids generating harmful high shear stress and enabling the distribution of gas and nutrients, but supplementing with DS further prevents adhesion among aggregates [23]. This combination should lead to the harvesting of high-quantity and high-quality hPSCs with homogeneous-sized hPSC aggregates, thus limiting various differentiation trends caused by a variety of the sizes of aggregates [18, 19]. Therefore, it is necessary to understand the molecular mechanism of DS in 3D suspension culture of hPSCs.

We and others have shown that DS is effective in controlling the size of the hPSC aggregates in 3D suspension culture conditions [23, 34, 37]. However, the molecular changes after DS treating on hPSCs are hardly understood. In our previous research, we have compared the transcriptome of hPSCs after treatment with DS or control groups, and many genes and pathways related to cell adhesion were found to be expressed differentially [23]; this guided us to further explore the molecular mechanisms. In the present study, we revealed the regulatory mechanism of DS treatment in controlling the aggregate size of hPSCs in 3D suspension culture conditions. First, the expression of CAMs such as ICAM1 was down-regulated after DS treatment. In addition, DS stimulated hPSCs to activate the expression of Wnt ligands and receptors, further promoting the expression of canonical Wnt signaling pathway target genes such as Twist1, Snai2, MMP3, and MMP7, consequently suppressing the expression of E-cad (Fig. 7D).

A number of reports revealed that the CAMs-mediated cohesive interaction among cells contributed significantly to the self-renewal and the pluripotent state of hPSCs [52, 53]. Moreover, the strong expression of CAMs such as E-cad is also related to somatic cell reprogramming of iPSCs. The enhancement of E-cad could promote the reprogramming efficiency of iPSCs and even replace the demand for OCT4 during the iPSC reprogramming [54, 55]. E-cad has also been used as an undifferentiated marker to identify pluripotent stem cells [56], indicating the importance of CAMs in regulating the pluripotency and stemness of hPSCs [57]. The expression of E-cad directly affects the differentiation potential and proliferation rate of hPSCs [58]. However, after DS treatment, the multi-lineage differentiation ability and proliferative capacity of hPSC aggregates have not been obviously altered [23], suggesting that the regulation of E-cad and other CAMs by DS impacts on aggregate adhesion rather than the stemness of hPSCs. The expression of TIMP2/3 is also bound up with E-cad. They complex with metalloproteinases such as MMP families (e.g., MMP3/7) and irreversibly inactivate MMP3/7 by binding to their catalytic zinc cofactor. However, the expression of TIMP2/3 was down-regulated significantly with DS treatment, resulting in the over-expression of MMP3/7 genes, and further interfering with the expression of E-cad on hPSCs [59].

ICAM1 is a transmembrane glycoprotein in the immunoglobulin superfamily, regulating signal transduction and cell–cell adhesion [60]. ICAM1 has been reported to mediate adhesion-dependent cell–cell interactions and to play an important role in regulating the size of tumors and the spherical sizes of cancer cell lines in vivo and in vitro [43, 61, 62]. Furthermore, ICAM1 is also expressed on stem cells, including bone marrow mesenchymal stem cells, periodontal ligament stem cells, and adipose stem cells [63, 64]. However, the function of ICAM1 in hPSCs has not been investigated. In this study, we first revealed that the expression of ICAM1 was important for the adhesion of hPSCs during the culture. Furthermore, the expression of ICAM1 was suppressed by DS treatment of hPSCs in 3D suspension conditions and the inhibition of ICAM1 prevented the adhesion among the aggregates of hPSCs, and finally reduced the heterogeneity of aggregate diameters.

The insights acquired in the current study increased our understanding of how DS prevented the adhesion among the aggregates of hPSCs. Previous studies suggested that the surface charge of CHO cells was altered by DS [35] and thus prevented the adhesion between CHO cells. Taken together, our results suggest that DS treatment prevented the adhesion through suppressing the expression of CAMs coupled with activating the canonical Wnt signaling pathway to suppress the expression of E-cad, thus co-regulating the size formation of the aggregates of hPSCs in 3D culture system.

## Conclusion

In the present study, we demonstrated that DS controlled the size of hPSC aggregates in 3D suspension culture through co-regulating the expression of CAMs, E-cad and ICAM1. By suppressing the expression of ICAM1, together with activating the canonical Wnt signaling pathway for further suppressing the expression of E-cad, the cell adhesion ability of hPSCs was attenuated and the sizes of aggregates were decreased. This in turn will allow for the retrieval of high-quality hPSCs and for enhancing the integrated differentiation process. The aggregate large-scale expansion strategy of hPSCs with the addition of DS is highly promising, and the results of this study may be helpful for improving the use of DS in 3D suspension cultures of hPSC in the future.

## Supplementary Information

**Additional file 1: Table S1**. Primers used. Including the sequence of primers used in qRT-PCR analysis.

**Additional file 2: Table S2**. Antibodies used. Including the formation of antibodies used in IF and WB analysis.

**Additional file 3: Fig. S1**. The evaluation of the pluripotency of H9 and hiPSCs. (A) Representative images of H9 and hiPSC colonies. Scale bars = 200 μm. (B) Flow cytometry analysis of H9 and hiPSCs for the expressions of OCT4, SSEA4, and TRA-1-81. (C) Immunofluorescence analysis of TRA-1-60 (red) on colonies of H9 and hiPSCs, respectively; the nuclei were stained with DAPI (blue). Scale bars = 100 μm. (D) Immunofluorescence analysis of NANOG (green) and SSEA4 (red) on colonies of H9 and hiPSCs, respectively; the nuclei were stained with DAPI (blue). Scale bars = 100 μm.

**Additional file 4: Fig. S2**. The effect of DS on hiPSC aggregates. (A) Representative images of the hiPSC aggregates on day 5 after the treatment with 100 μg/ml DS. Scale bar = 200 μm. (B) Comparison of average diameter of the hiPSC aggregates on day 5 after the treatment with 100 μg/ml DS. (C) Diameter distribution of the hiPSC aggregates treated with or without DS on day 5. (D) Comparison of the cell numbers after 5 days of culture.

**Additional file 5: Fig. S3**. Cell counting assay 24 hours after the treatment with DS and Wnt inhibitor. (A) Representative images of the cells treated with or without DS, supplemented with 5 μM Wnt inhibitor IWR-1 or IWP2 24 hours after the treatment. Scale bar = 200 μm. (B) Comparison of the cell numbers 24 hours after inoculation.

**Additional file 6: Fig. S4**. Rescue assay with Wnt inhibitors in 3D suspension culture condition. (A) Representative images of the aggregates treated with or without DS plus 5 μM Wnt inhibitor IWR-1 or IWP2 on day 5 after the treatment. Scale bar = 200 μm. (B) Comparison of average diameter of the H9 aggregates on day 5 after the treatment. (C) Diameter distribution of the H9 aggregates on day 5 after the treatment. (D) Comparison of the cell numbers after 5 days of culture. (E) Gene expression analysis by qRT-PCR for E-cad.

**Additional file 7: Fig. S5**. Simulation of the effect of DS by replacing DS with ICAM1 inhibitor and Wnt agonist. (A) Representative images of the aggregates treated with or without 10 μM A-205804 plus 3 μM CHIR99021 or 10 ng/ml WNT3A on day 5 after the treatment. Scale bar = 200 μm. (B) Gene expression analysis by qRT-PCR for pluripotent genes, OCT4, SOX2, and NANOG. (C) Gene expression analysis by qRT-PCR for genes SOX17, BRACHYURY, and GATA4 of mesoendoderm germ layers.

## Data Availability

The data that support the findings of this study are available from the corresponding author upon reasonable request.

## Abbreviations

hPSC: Human pluripotent stem cells
DS: Dextran sulfate
CAMs: Cellular adhesion molecules
E-cad: E-cadherin
ICAM1: Intercellular adhesion molecule 1
hESCs: Human embryonic stem cells
hiPSCs: Human-induced pluripotent stem cells
2D: Two-dimensional
3D: Three-dimensional
GMP: Good manufacturing practice
CHO: Chinese hamster ovary
KSR: Knockout serum replacement

## References

[1] Thomson JA, Itskovitz-Eldor J, Shapiro SS, Waknitz MA, Swiergiel JJ, Marshall VS, Jones JM. Embryonic stem cell lines derived from human blastocysts. Science. 1998;282:1145–7. 9804556 10.1126/science.282.5391.1145

[2] Takahashi K, Tanabe K, Ohnuki M, Narita M, Ichisaka T, Tomoda K, Yamanaka S. Induction of pluripotent stem cells from adult human fibroblasts by defined factors. Cell. 2007;131:861–72. 18035408 10.1016/j.cell.2007.11.019

[3] Fox IJ, Daley GQ, Goldman SA, Huard J, Kamp TJ, Trucco M. Stem cell therapy Use of differentiated pluripotent stem cells as replacement therapy for treating disease. Science. 2014;345:1247391. 25146295 10.1126/science.1247391 PMC4329726

[4] Villa-Diaz LG, Ross AM, Lahann J, Krebsbach PH. Concise review: the evolution of human pluripotent stem cell culture: from feeder cells to synthetic coatings. Stem Cells. 2013;31:1–7. 23081828 10.1002/stem.1260 PMC3537180

[5] Chen KG, Mallon BS, McKay RD, Robey PG. Human pluripotent stem cell culture: considerations for maintenance, expansion, and therapeutics. Cell Stem Cell. 2014;14:13–26. 24388173 10.1016/j.stem.2013.12.005 PMC3915741

[6] Forbes SJ, Gupta S, Dhawan A. Cell therapy for liver disease: from liver transplantation to cell factory. J Hepatol. 2015;62:S157–69. 25920085 10.1016/j.jhep.2015.02.040

[7] Pareja E, Gómez-Lechón MJ, Tolosa L. Induced pluripotent stem cells for the treatment of liver diseases: challenges and perspectives from a clinical viewpoint. Ann Transl Med. 2020;8:566. 32775367 10.21037/atm.2020.02.164 PMC7347783

[8] Lock LT, Tzanakakis ES. Stem/Progenitor cell sources of insulin-producing cells for the treatment of diabetes. Tissue Eng. 2007;13:1399–412. 17550339 10.1089/ten.2007.0047

[9] Zweigerdt R. Large scale production of stem cells and their derivatives. Adv Biochem Eng Biotechnol. 2009;114:201–35. 19513633 10.1007/10_2008_27

[10] Lei Y, Schaffer DV. A fully defined and scalable 3D culture system for human pluripotent stem cell expansion and differentiation. Proc Natl Acad Sci USA. 2013;110:E5039–48. 24248365 10.1073/pnas.1309408110 PMC3876251

[11] Chen VC, Couture SM, Ye J, Lin Z, Hua G, Huang HI, Wu J, Hsu D, Carpenter MK, Couture LA. Scalable GMP compliant suspension culture system for human ES cells. Stem Cell Res. 2012;8:388–402. 22459095 10.1016/j.scr.2012.02.001

[12] Fan Y, Zhang F, Tzanakakis ES. Engineering xeno-free microcarriers with recombinant vitronectin, albumin and UV Irradiation for human pluripotent stem cell bioprocessing. ACS Biomater Sci Eng. 2017;3:1510–8. 28989958 10.1021/acsbiomaterials.6b00253 PMC5630174

[13] Fattahi P, Rahimian A, Slama MQ, Gwon K, Gonzalez-Suarez AM, Wolf J, Baskaran H, Duffy CD, Stybayeva G, Peterson QP, Revzin A. Core-shell hydrogel microcapsules enable formation of human pluripotent stem cell spheroids and their cultivation in a stirred bioreactor. Sci Rep. 2021;11:7177. 33785778 10.1038/s41598-021-85786-2 PMC8010084

[14] Li X, Ma R, Gu Q, Liang L, Wang L, Zhang Y, Wang X, Liu X, Li Z, Fang J, Wu J, Wang Y, Li W, Hu B, Wang L, Zhou Q, Hao J. A fully defined static suspension culture system for large-scale human embryonic stem cell production. Cell Death Dis. 2018;9:892. 30166524 10.1038/s41419-018-0863-8 PMC6117302

[15] Wang Y, Chou BK, Dowey S, He C, Gerecht S, Cheng L. Scalable expansion of human induced pluripotent stem cells in the defined xeno-free E8 medium under adherent and suspension culture conditions. Stem Cell Res. 2013;11:1103–16. 23973800 10.1016/j.scr.2013.07.011 PMC4628790

[16] Nath SC, Nagamori E, Horie M, Kino-Oka M. Culture medium refinement by dialysis for the expansion of human induced pluripotent stem cells in suspension culture. Bioprocess Biosyst Eng. 2017;40:123–31. 27638317 10.1007/s00449-016-1680-z

[17] Borys BS, Dang T, So T, Rohani L, Revay T, Walsh T, Thompson M, Argiropoulos B, Rancourt DE, Jung S, Hashimura Y, Lee B, Kallos MS. Overcoming bioprocess bottlenecks in the large-scale expansion of high-quality hiPSC aggregates in vertical-wheel stirred suspension bioreactors. Stem Cell Res Ther. 2021;12:55. 33436078 10.1186/s13287-020-02109-4 PMC7805206

[18] Bauwens CL, Peerani R, Niebruegge S, Woodhouse KA, Kumacheva E, Husain M, Zandstra PW. Control of human embryonic stem cell colony and aggregate size heterogeneity influences differentiation trajectories. Stem Cells. 2008;26:2300–10. 18583540 10.1634/stemcells.2008-0183

[19] Farzaneh Z, Najarasl M, Abbasalizadeh S, Vosough M, Baharvand H. Developing a cost-effective and scalable production of human hepatic competent endoderm from size-controlled pluripotent stem cell aggregates. Stem Cells Dev. 2018;27:262–74. 29298619 10.1089/scd.2017.0074

[20] Lee G, Kim H, Park JY, Kim G, Han J, Chung S, Yang JH, Jeon JS, Woo DH, Han C, Kim SK, Park HJ, Kim JH. Generation of uniform liver spheroids from human pluripotent stem cells for imaging-based drug toxicity analysis. Biomaterials. 2021;269:120529. 33257114 10.1016/j.biomaterials.2020.120529

[21] Wu J, Rostami MR, Cadavid Olaya DP, Tzanakakis ES. Oxygen transport and stem cell aggregation in stirred-suspension bioreactor cultures. PLoS ONE. 2014;9:e102486. 25032842 10.1371/journal.pone.0102486 PMC4102498

[22] Edmondson R, Broglie JJ, Adcock AF, Yang L. Three-dimensional cell culture systems and their applications in drug discovery and cell-based biosensors. Assay Drug Dev Technol. 2014;12:207–18. 24831787 10.1089/adt.2014.573 PMC4026212

[23] Tang X, Wu H, Xie J, Wang N, Chen Q, Zhong Z, Qiu Y, Wang J, Li X, Situ P, Lai L, Zern MA, Chen H, Duan Y. The combination of dextran sulphate and polyvinyl alcohol prevents excess aggregation and promotes proliferation of pluripotent stem cells in suspension culture. Cell Prolif. 2021;54:e13112. 34390064 10.1111/cpr.13112 PMC8450127

[24] Bauwens CL, Toms D, Ungrin M. Aggregate size optimization in microwells for suspension-based cardiac differentiation of human pluripotent stem cells. J Vis Exp. 2016;115:54. 10.3791/54308 PMC5092056 27768032

[25] Nampe D, Joshi R, Keller K, Zur Nieden NI, Tsutsui H. Impact of fluidic agitation on human pluripotent stem cells in stirred suspension culture. Biotechnol Bioeng. 2017;114:2109–20. 28480972 10.1002/bit.26334

[26] Manstein F, Ullmann K, Kropp C, Halloin C, Triebert W, Franke A, Farr CM, Sahabian A, Haase A, Breitkreuz Y, Peitz M, Brüstle O, Kalies S, Martin U, Olmer R, Zweigerdt R. High density bioprocessing of human pluripotent stem cells by metabolic control and in silico modeling. Stem Cells Transl Med. 2021;10:1063–80. 33660952 10.1002/sctm.20-0453 PMC8235132

[27] Xu S, Gavin J, Jiang R, Chen H. Bioreactor productivity and media cost comparison for different intensified cell culture processes. Biotechnol Prog. 2017;33:867–78. 27977910 10.1002/btpr.2415

[28] Donini M, Marusic C. Current state-of-the-art in plant-based antibody production systems. Biotechnol Lett. 2019;41:335–46. 30684155 10.1007/s10529-019-02651-z

[29] Lei X, Deng Z, Zhang H, Zhao H, Zhou J, Liu S, Chen Q, Ning L, Cao Y, Wang X, Zhang X, Duan E. Rotary suspension culture enhances mesendoderm differentiation of embryonic stem cells through modulation of Wnt/β-catenin pathway. Stem Cell Rev Rep. 2014;10:526–38. 24793926 10.1007/s12015-014-9511-6

[30] Stolberg S, McCloskey KE. Can shear stress direct stem cell fate? Biotechnol Prog. 2009;25:10–9. 19197983 10.1002/btpr.124

[31] Horiguchi I, Torizal FG, Nagate H, Inose H, Inamura K, Hirata O, Hayashi H, Horikawa M, Sakai Y. Protection of human induced pluripotent stem cells against shear stress in suspension culture by Bingham plastic fluid. Biotechnol Prog. 2021;37:e3100. 33169533 10.1002/btpr.3100 PMC8244041

[32] Horiguchi I, Sakai Y. Serum replacement with albumin-associated lipids prevents excess aggregation and enhances growth of induced pluripotent stem cells in suspension culture. Biotechnol Prog. 2016;32:1009–16. 27193385 10.1002/btpr.2301

[33] Ibuki M, Horiguchi I, Sakai Y. A novel tool for suspension culture of human induced pluripotent stem cells: Lysophospholipids as a cell aggregation regulator. Regen Ther. 2019;12:74–82. 31890769 10.1016/j.reth.2019.03.008 PMC6933451

[34] Lipsitz YY, Tonge PD, Zandstra PW. Chemically controlled aggregation of pluripotent stem cells. Biotechnol Bioeng. 2018;115:2061–6. 29679475 10.1002/bit.26719 PMC6055717

[35] Dee KU, Shuler ML, Wood HA. Inducing single-cell suspension of BTI-TN5B1-4 insect cells: I. The use of sulfated polyanions to prevent cell aggregation and enhance recombinant protein production. Biotechnol Bioeng. 1997;54:191–205. 18634086 10.1002/(SICI)1097-0290(19970505)54:3<191::AID-BIT1>3.0.CO;2-A

[36] Hyoung Park J, Sin Lim M, Rang Woo J, Won Kim J, Min G, Lee. The molecular weight and concentration of dextran sulfate affect cell growth and antibody production in CHO cell cultures. Biotechnol Prog. 2016;32:1113–22. 27114230 10.1002/btpr.2287

[37] Nogueira DES, Rodrigues CAV, Carvalho MS, Miranda CC, Hashimura Y, Jung S, Lee B, Cabral JMS. Strategies for the expansion of human induced pluripotent stem cells as aggregates in single-use Vertical-Wheel™ bioreactors. J Biol Eng. 2019;13:74. 31534477 10.1186/s13036-019-0204-1 PMC6744632

[38] Ai Z, Niu B, Duan K, Si C, Wang S, Xiang L, Zhu X, Zhu Q, Feng C, Yin Y, Zhao S, Kong R, Ji W, Li T. Modulation of Wnt and Activin/Nodal supports efficient derivation, cloning and suspension expansion of human pluripotent stem cells. Biomaterials. 2020;249:120015. 32311594 10.1016/j.biomaterials.2020.120015

[39] Wei RR, Sun DN, Yang H, Yan J, Zhang X, Zheng XL, Fu XH, Geng MY, Huang X, Ding J. CTC clusters induced by heparanase enhance breast cancer metastasis. Acta Pharmacol Sin. 2018;39:1326–37. 29417941 10.1038/aps.2017.189 PMC6289387

[40] Lipsitz YY, Woodford C, Yin T, Hanna JH, Zandstra PW. Modulating cell state to enhance suspension expansion of human pluripotent stem cells. Proc Natl Acad Sci USA. 2018;115:6369–74. 29866848 10.1073/pnas.1714099115 PMC6016797

[41] Fico F, Santamaria-Martínez A. TGFBI modulates tumour hypoxia and promotes breast cancer metastasis. Mol Oncol. 2020;14:3198–210. 33080107 10.1002/1878-0261.12828 PMC7718944

[42] Sart S, Bejoy J, Li Y. Characterization of 3D pluripotent stem cell aggregates and the impact of their properties on bioprocessing. Process Biochem. 2017;59:276–88.

[43] Li C, Liu S, Yan R, Han N, Wong KK, Li L. CD54-NOTCH1 axis controls tumor initiation and cancer stem cell functions in human prostate cancer. Theranostics. 2017;7:67–80. 28042317 10.7150/thno.16752 PMC5196886

[44] Azarin SM, Lian X, Larson EA, Popelka HM, de Pablo JJ, Palecek SP. Modulation of Wnt/β-catenin signaling in human embryonic stem cells using a 3-D microwell array. Biomaterials. 2012;33:2041–9. 22177620 10.1016/j.biomaterials.2011.11.070 PMC3259207

[45] Chan SC, Hajarnis SS, Vrba SM, Patel V, Igarashi P. Hepatocyte nuclear factor 1β suppresses canonical Wnt signaling through transcriptional repression of lymphoid enhancer-binding factor 1. J Biol Chem. 2020;295:17560–72. 33453998 10.1074/jbc.RA120.015592 PMC7762946

[46] Heuberger J, Birchmeier W. Interplay of cadherin-mediated cell adhesion and canonical Wnt signaling. Cold Spring Harb Perspect Biol. 2010;2:a002915. 20182623 10.1101/cshperspect.a002915 PMC2828280

[47] Konze SA, van Diepen L, Schröder A, Olmer R, Möller H, Pich A, Weißmann R, Kuss AW, Zweigerdt R, Buettner FF. Cleavage of E-cadherin and β-catenin by calpain affects Wnt signaling and spheroid formation in suspension cultures of human pluripotent stem cells. Mol Cell Proteomics. 2014;13:990–1007. 24482122 10.1074/mcp.M113.033423 PMC3977196

[48] Rehakova D, Souralova T, Koutna I. Clinical-grade human pluripotent stem cells for cell therapy: characterization strategy. Int J Mol Sci. 2020;21:2435. 32244538 10.3390/ijms21072435 PMC7177280

[49] Chen KG, Mallon BS, Park K, Robey PG, McKay RDG, Gottesman MM, Zheng W. Pluripotent stem cell platforms for drug discovery. Trends Mol Med. 2018;24:805–20. 30006147 10.1016/j.molmed.2018.06.009 PMC6117164

[50] Soares FA, Chandra A, Thomas RJ, Pedersen RA, Vallier L, Williams DJ. Investigating the feasibility of scale up and automation of human induced pluripotent stem cells cultured in aggregates in feeder free conditions. J Biotechnol. 2014;173:53–8. 24440272 10.1016/j.jbiotec.2013.12.009 PMC3969287

[51] Kropp C, Kempf H, Halloin C, Robles-Diaz D, Franke A, Scheper T, Kinast K, Knorpp T, Joos TO, Haverich A, Martin U, Zweigerdt R, Olmer R. Impact of feeding strategies on the scalable expansion of human pluripotent stem cells in single-use stirred tank bioreactors. Stem Cells Transl Med. 2016;5:1289–301. 27369897 10.5966/sctm.2015-0253 PMC5031176

[52] Chen T, Yuan D, Wei B, Jiang J, Kang J, Ling K, Gu Y, Li J, Xiao L, Pei G. E-cadherin-mediated cell-cell contact is critical for induced pluripotent stem cell generation. Stem Cells. 2010;28:1315–25. 20521328 10.1002/stem.456

[53] Rowland TJ, Miller LM, Blaschke AJ, Doss EL, Bonham AJ, Hikita ST, Johnson LV, Clegg DO. Roles of integrins in human induced pluripotent stem cell growth on Matrigel and vitronectin. Stem Cells Dev. 2010;19:1231–40. 19811096 10.1089/scd.2009.0328

[54] Redmer T, Diecke S, Grigoryan T, Quiroga-Negreira A, Birchmeier W, Besser D. E-cadherin is crucial for embryonic stem cell pluripotency and can replace OCT4 during somatic cell reprogramming. EMBO Rep. 2011;12:720–6. 21617704 10.1038/embor.2011.88 PMC3128971

[55] Hansson J, Rafiee MR, Reiland S, Polo JM, Gehring J, Okawa S, Huber W, Hochedlinger K, Krijgsveld J. Highly coordinated proteome dynamics during reprogramming of somatic cells to pluripotency. Cell Rep. 2012;2:1579–92. 23260666 10.1016/j.celrep.2012.10.014 PMC4438680

[56] Yao S, Chen S, Clark J, Hao E, Beattie GM, Hayek A, Ding S. Long-term self-renewal and directed differentiation of human embryonic stem cells in chemically defined conditions. Proc Natl Acad Sci USA. 2006;103:6907–12. 16632596 10.1073/pnas.0602280103 PMC1458992

[57] Li L, Bennett SA, Wang L. Role of E-cadherin and other cell adhesion molecules in survival and differentiation of human pluripotent stem cells. Cell Adh Migr. 2012;6:59–70. 22647941 10.4161/cam.19583 PMC3364139

[58] Yu L, Li J, Hong J, Takashima Y, Fujimoto N, Nakajima M, Yamamoto A, Dong X, Dang Y, Hou Y, Yang W, Minami I, Okita K, Tanaka M, Luo C, Tang F, Chen Y, Tang C, Kotera H, Liu L. Low cell-matrix adhesion reveals two subtypes of human pluripotent stem cells. Stem Cell Rep. 2018;11:142–56. 10.1016/j.stemcr.2018.06.003 PMC6067523 30008324

[59] Nie G, Wen X, Liang X, Zhao H, Li Y, Lu J. Additional evidence supports association of common genetic variants in MMP3 and TIMP2 with increased risk of chronic Achilles tendinopathy susceptibility. J Sci Med Sport. 2019;22:1074–8. 31208828 10.1016/j.jsams.2019.05.021

[60] Howard K, Lo KK, Ao L, Gamboni F, Edil BH, Schulick R, Barnett CC Jr. Intercellular adhesion molecule-1 mediates murine colon adenocarcinoma invasion. J Surg Res. 2014;187:19–23. 24360118 10.1016/j.jss.2013.11.001 PMC4844553

[61] Lv G, Fan J. Silencing ICAM-1 reduces the adhesion of vascular endothelial cells in mice with immunologic contact urticaria. Gene. 2020;760: 144965. 32687948 10.1016/j.gene.2020.144965

[62] Liu S, Li N, Yu X, Xiao X, Cheng K, Hu J, Wang J, Zhang D, Cheng S, Liu S. Expression of intercellular adhesion molecule 1 by hepatocellular carcinoma stem cells and circulating tumor cells. Gastroenterology. 2013;144:1031-1041.e10. 23376424 10.1053/j.gastro.2013.01.046

[63] Assis AC, Carvalho JL, Jacoby BA, Ferreira RL, Castanheira P, Diniz SO, Cardoso VN, Goes AM, Ferreira AJ. Time-dependent migration of systemically delivered bone marrow mesenchymal stem cells to the infarcted heart. Cell Transplant. 2010;19:219–30. 19906330 10.3727/096368909X479677

[64] Sununliganon L, Singhatanadgit W. Highly osteogenic PDL stem cell clones specifically express elevated levels of ICAM1, ITGB1 and TERT. Cytotechnology. 2012;64:53–63. 21866310 10.1007/s10616-011-9390-5 PMC3261450

