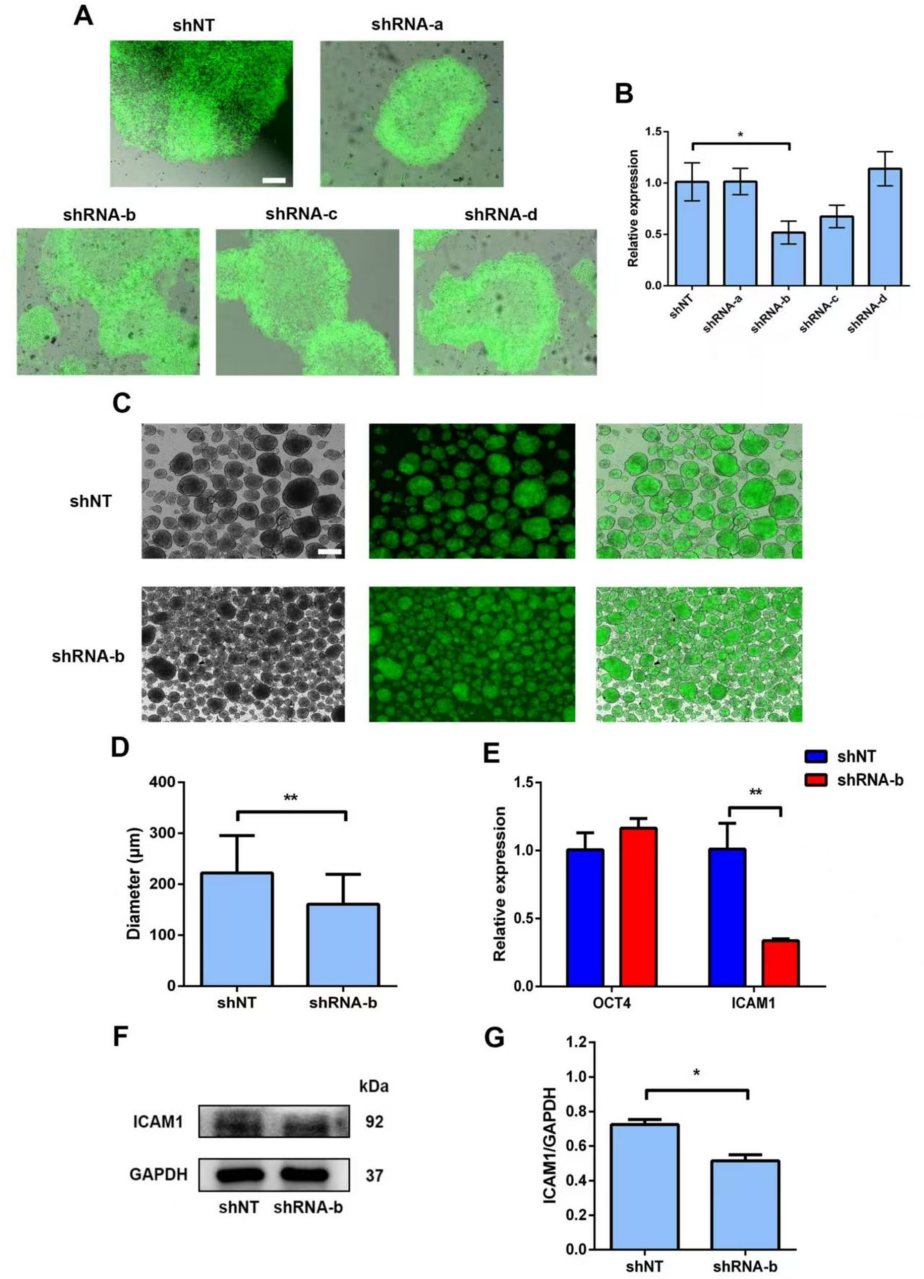# Correction: Dextran sulfate prevents excess aggregation of human pluripotent stem cells in 3D culture by inhibiting ICAM1 expression coupled with down-regulating E-cadherin through activating the Wnt signaling pathway

**DOI:** 10.1186/s13287-025-04530-z

**Published:** 2025-07-26

**Authors:** Haibin Wu, Xianglian Tang, Yiyu Wang, Ning Wang, Qicong Chen, Jinghe Xie, Shoupei Liu, Zhiyong Zhong, Yaqi Qiu, Ping Situ, Mark A. Zern, Jue Wang, Honglin Chen, Yuyou Duan

**Affiliations:** 1 Laboratory of Stem Cells and Translational Medicine, Institutes for Life Sciences, School of Medicine, South China University of Technology, No. 382 Waihuan East Road, Suite 406, Higher Education Mega Center, Guangzhou, 510006 People’s Republic of China; 2 School of Biomedical Sciences and Engineering, South China University of Technology, Guangzhou International Campus, Guangzhou, 510180 People’s Republic of China; 3 Guangxi Key Laboratory of Reproductive Health and Birth Defects Prevention, Guangxi Health Commission Key Laboratory of Precise Diagnosis and Treatment of Genetic Diseases, Maternal and Child Health Hospital of Guangxi Zhuang Autonomous Region, Nanning, 530003 Guangxi People’s Republic of China; 4 Genetic and Metabolic Central Laboratory, Guangxi Birth Defects Research and Prevention Institute, Nanning, 530003 Guangxi People’s Republic of China; 5 Department of Internal Medicine, University of California Davis Medical Center,, Sacramento, CA 95817 USA; 6 National Engineering Research Center for Tissue Restoration and Reconstruction, South China University of Technology, Guangzhou, 510180 People’s Republic of China; 7 Guangdong Provincial Key Laboratory of Biomedical Engineering, South China University of Technology, Guangzhou, 510180 People’s Republic of China; 8 Key Laboratory of Biomedical Materials and Engineering of the Ministry of Education, South China University of Technology, Guangzhou, 510180 People’s Republic of China

**Stem Cell Research & Therapy (2022) 13:218**

10.1186/s13287-022-02890-4

Following publication of this study [1], the authors realised that the images in Fig. 5A for shNT, shRNA-a and shRNA-b were inadvertently misused during the preparation of this article. Upon reviewing the original records, the authors have now provided the correct images for shNT, shRNA-a and shRNA-b in Fig. 5A.

This correction does not affect the findings or conclusions of this article. The authors sincerely apologise for this oversight which may cause confusion or inconvenience.

**Figure:**