# Supplementary material for: Dextran sulfate prevents excess aggregation of human pluripotent stem cells in 3D culture by inhibiting ICAM1 expression coupled with down-regulating E-cadherin through activating the Wnt signaling pathway
Source: Stem Cell Res Ther. 2022 May 26;13:218. doi: 10.1186/s13287-022-02890-4 (PMC9137216; doi:10.1186/s13287-022-02890-4)
Supplement: Supplementary file 1 — Additional file 1: Table S1. Primers used. Including the sequence of primers used in qRT-PCR analysis. [file 13287_2022_2890_MOESM1_ESM.docx]

| **Supplementary Table 1- Primers used** | | |
| --- | --- | --- |
| **Gene** | **Forward** | **Reverse** |
| **ICAM1** | **GACTAAGCCAAGAGGAAGGAGCAA** | **TCAGCATACCCAATAGGCAGCAAG** |
| **E-CAD** | **CCCACCACGTACAAGGGTC** | **CTGGGGTATTGGGGGCATC** |
| **TIMP2** | **GTGCAACTTCGTGGAGAGGT** | **CAGGTAGTAGCAGGACTTGATCTTG** |
| **TIMP3** | **ACAGGCGTTTTGCAATGCA** | **GGGTTGCCATAAATGTCGTTTC** |
| **P-CAD** | **AGGAGACAGGCTGGTTGTTG** | **GTTCATGGGGTCCTCCACTG** |
| **ITGB2** | **TGCGTCCTCTCTCAGGAGTG** | **GGTCCATGATGTCGTCAGCC** |
| **ITGA5** | **GCGTGCCCAAGGGGAACCTC** | **AGCAGGGGTGCCCCTACCAG** |
| **ITGA7** | **GCTGTGAAGTCCCTGGAAGTGATT** | **GCATCTCGGAGCATCAAGTTCTT** |
| **TGFβi** | **TGCTCCCACAAATGAAGCCT** | **GCCTCCGCTAACCAGGATTT** |
| **VTN** | **TGCTGGCATGGGTTGCT** | **GTTCATGGACAGTGGCATTGTT** |
| **ECM1** | **CTGCTGTGACCTGCCATTTC** | **TCCCCAGGACTCAGGTAACA** |
| **PECAM1** | **CCAGTGTCCCCAGAAGCAAA** | **TCCGATGACAACCACTGCAA** |
| **WNT4** | **GCTGGAGAAGTGCGGCTGTGA** | **CCACAAACGACTGTGAGAAGGC** |
| **WNT7B** | **AGAAGACCGTCTTCGGGCAAGA** | **AGTTGCTCAGGTTCCCTTGGCT** |
| **WNT8A** | **CATCGAGGAGTGCAAGTTCCAG** | **GCAGAGCTGATAGCATGTATGAAG** |
| **WNT10B** | **CTCGGGATTTCTTGGATTCCAGG** | **GCCATGACACTTGCATTTCCGC** |
| **FZD5** | **TGGAACGCTTCCGCTATCCTGA** | **GGTCTCGTAGTGGATGTGGTTG** |
| **FZD8** | **GCTCTACAACCGCGTCAAGACA** | **AAGGTGGACACGAAGCAGAGCA** |
| **LEF1** | **CTACCCATCCTCACTGTCAGTC** | **GGATGTTCCTGTTTGACCTGAGG** |
| **SNAI1** | **TGCCCTCAAGATGCACATCCGA** | **GGGACAGGAGAAGGGCTTCTC** |
| **SNAI2** | **ATCTGCGGCAAGGCGTTTTCCA** | **GAGCCCTCAGATTTGACCTGTC** |
| **SNAI3** | **TGCACCTGCAAGATCTGTGGCA** | **AAGGTTGGAGCGGTCGGCAAAG** |
| **SNAIL** | **TGCCCTCAAGATGCACATCCGA** | **GGGACAGGAGAAGGGCTTCTC** |
| **ADAM10** | **GAGGAGTGTACGTGTGCCAGTT** | **GACCACTGAAGTGCCTACTCCA** |
| **MMP3** | **CACTCACAGACCTGACTCGGTT** | **AAGCAGGATCACAGTTGGCTGG** |
| **MMP7** | **TCGGAGGAGATGCTCACTTCGA** | **GGATCAGAGGAATGTCCCATACC** |
| **TWIST1** | **GCCAGGTACATCGACTTCCTCT** | **TCCATCCTCCAGACCGAGAAGG** |
| **TWIST2** | **GCAAGATCCAGACGCTCAAGCT** | **ACACGGAGAAGGCGTAGCTGAG** |
| **GAPDH** | **GAAGATGGTGATGGGATTTC** | **GAAGGTGAAGGTCGGAGTC** |
