# Supplementary material for: Dextran sulfate prevents excess aggregation of human pluripotent stem cells in 3D culture by inhibiting ICAM1 expression coupled with down-regulating E-cadherin through activating the Wnt signaling pathway
Source: Stem Cell Res Ther. 2022 May 26;13:218. doi: 10.1186/s13287-022-02890-4 (PMC9137216; doi:10.1186/s13287-022-02890-4)
Supplement: Supplementary file 2 — Additional file 2: Table S2. Antibodies used. Including the formation of antibodies used in IF and WB analysis. [file 13287_2022_2890_MOESM2_ESM.docx]

| **Supplementary Table 2 – Antibodies used** | | | | |
| --- | --- | --- | --- | --- |
| **Primary antibody** | | | | |
| **Antibody** | **Company** | **Product code** | **Ig Species** | **Dilution** |
| **GAPDH** | **Abcam** | **ab128915** | **Rabbit** | **10000** |
| **CD54/ICAM1** | **CST** | **4915** | **Rabbit** | **1000** |
| **E-Cadherin** | **CST** | **3915** | **Rabbit** | **1000** |
| **Slug** | **CST** | **9585T** | **Rabbit** | **1000** |
| **Twist-1** | **R＆D** | **MAB6230** | **Rabbit** | **1000** |
| **β-catenin** | **Bioss** | **bs-23663R** | **Rabbit** | **500** |
| **MMP-3** | **Bioss** | **bs-0413R** | **Rabbit** | **500** |
| **LEF-1** | **Bioss** | **bs-1843R** | **Rabbit** | **500** |
| **Frizzled 8** | **Bioss** | **bs-13219R** | **Rabbit** | **500** |
| **WNT7B** | **Bioss** | **bs-6244R** | **Rabbit** | **500** |
| **PE Anti-Human OCT4 (OCT3) Antibody** | **STEM CELL** | **60093PE** |  | **5 μL for million cells** |
| **PE Anti-Human TRA-1-81 Antibody** | **STEM CELL** | **60065PE** |  | **5 μL for million cells** |
| **PE Anti-Human SSEA-4 Antibody** | **STEM CELL** | **60062PE** |  | **5 μL for million cells** |
| **PE mouse isotype-controlled antibody** | **BD** | **556650** |  | **20 μL for million cells** |
| **Nanog Rabbit Ab** | **CST** | **4903** | **Rabbit** | **1000** |
| **SSEA-4 Mouse mAb** | **CST** | **4755** | **Mouse** | **1000** |
| **TRA-1-60 Mouse mAb** | **CST** | **4746** | **Mouse** | **1000** |
|  |  |  |  |  |
| **Secondary antibody** | | | |  |
| **Secondary antibody** | **Company** | **Code number** | **Dilution** |  |
| **Alexa Fluor 594-conjugated goat anti-Rabbit IgG** | **CST** | **8889S** | **800** |  |
| **anti-rabbit IgG, HRP-linked antibody** | **CST** | **7074** | **3000** |  |
