# Supplementary figures and images for: Dextran sulfate prevents excess aggregation of human pluripotent stem cells in 3D culture by inhibiting ICAM1 expression coupled with down-regulating E-cadherin through activating the Wnt signaling pathway
Source: Stem Cell Res Ther. 2022 May 26;13:218. doi: 10.1186/s13287-022-02890-4 (PMC9137216; doi:10.1186/s13287-022-02890-4)

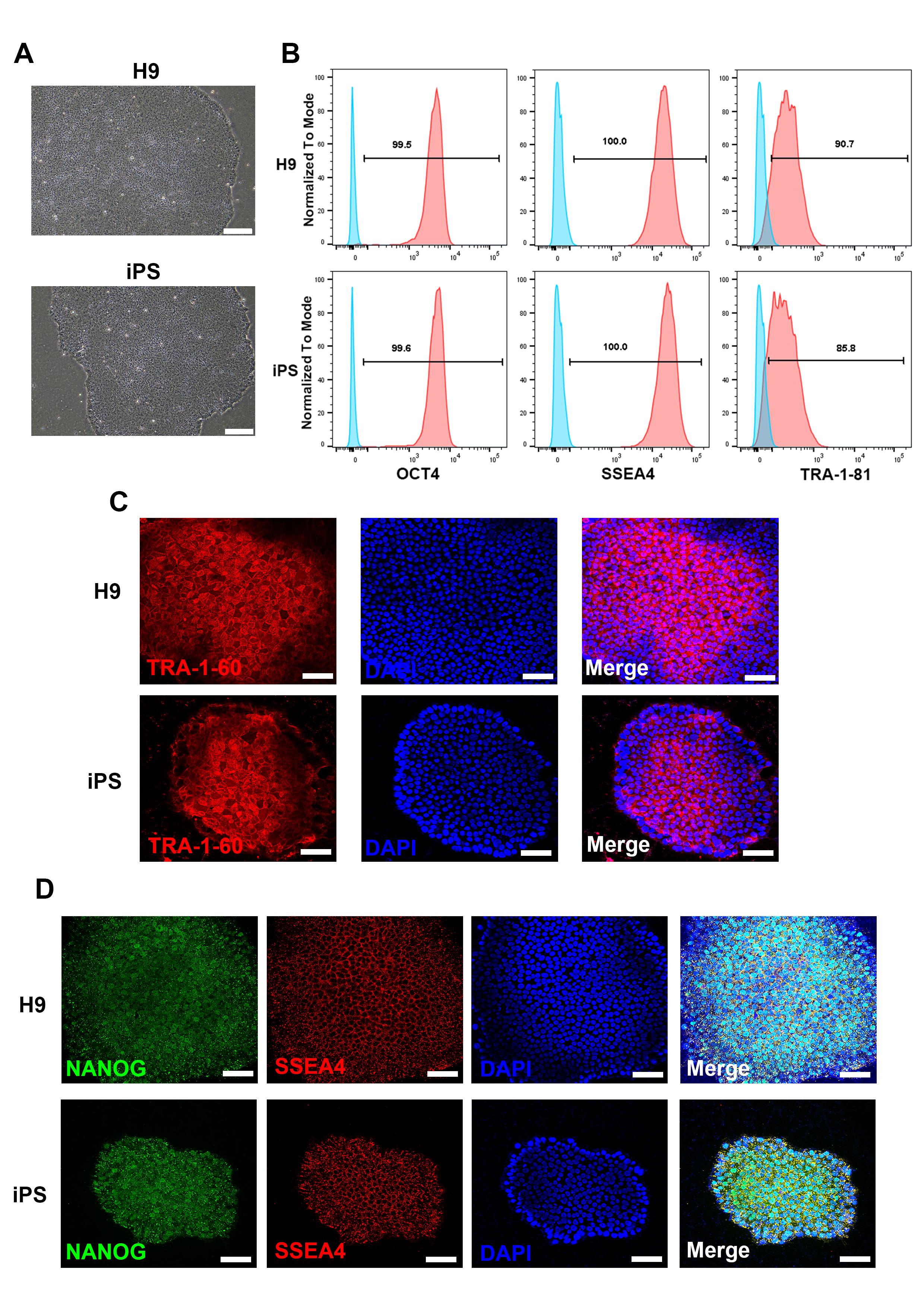

Supplement: Supplementary file 3 — Additional file 3: Fig. S1. The evaluation of the pluripotency of H9 and hiPSCs. (A) Representative images of H9 and hiPSC colonies. Scale bars = 200 μm. (B) Flow cytometry analysis of H9 and hiPSCs for the expressions of OCT4, SSEA4, and TRA-1-81. (C) Immunofluorescence analysis of TRA-1-60 (red) on colonies of H9 and hiPSCs, respectively; the nuclei were stained with DAPI (blue). Scale bars = 100 μm. (D) Immunofluorescence analysis of NANOG (green) and SSEA4 (red) on colonies of H9 and hiPSCs, respectively; the nuclei were stained with DAPI (blue). Scale bars = 100 μm. [file 13287_2022_2890_MOESM3_ESM.tif]

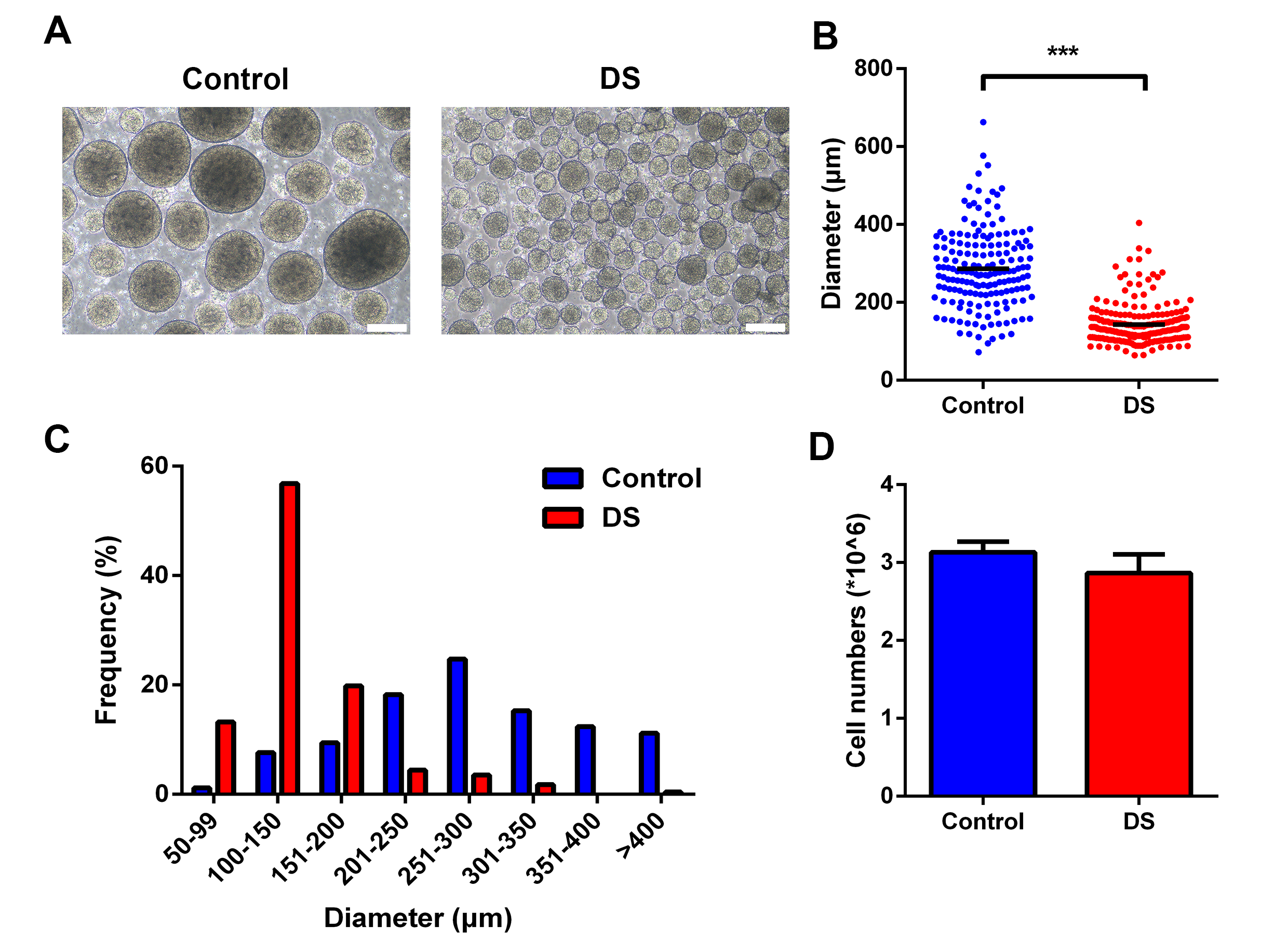

Supplement: Supplementary file 4 — Additional file 4: Fig. S2. The effect of DS on hiPSC aggregates. (A) Representative images of the hiPSC aggregates on day 5 after the treatment with 100 μg/ml DS. Scale bar = 200 μm. (B) Comparison of average diameter of the hiPSC aggregates on day 5 after the treatment with 100 μg/ml DS. (C) Diameter distribution of the hiPSC aggregates treated with or without DS on day 5. (D) Comparison of the cell numbers after 5 days of culture. [file 13287_2022_2890_MOESM4_ESM.tif]

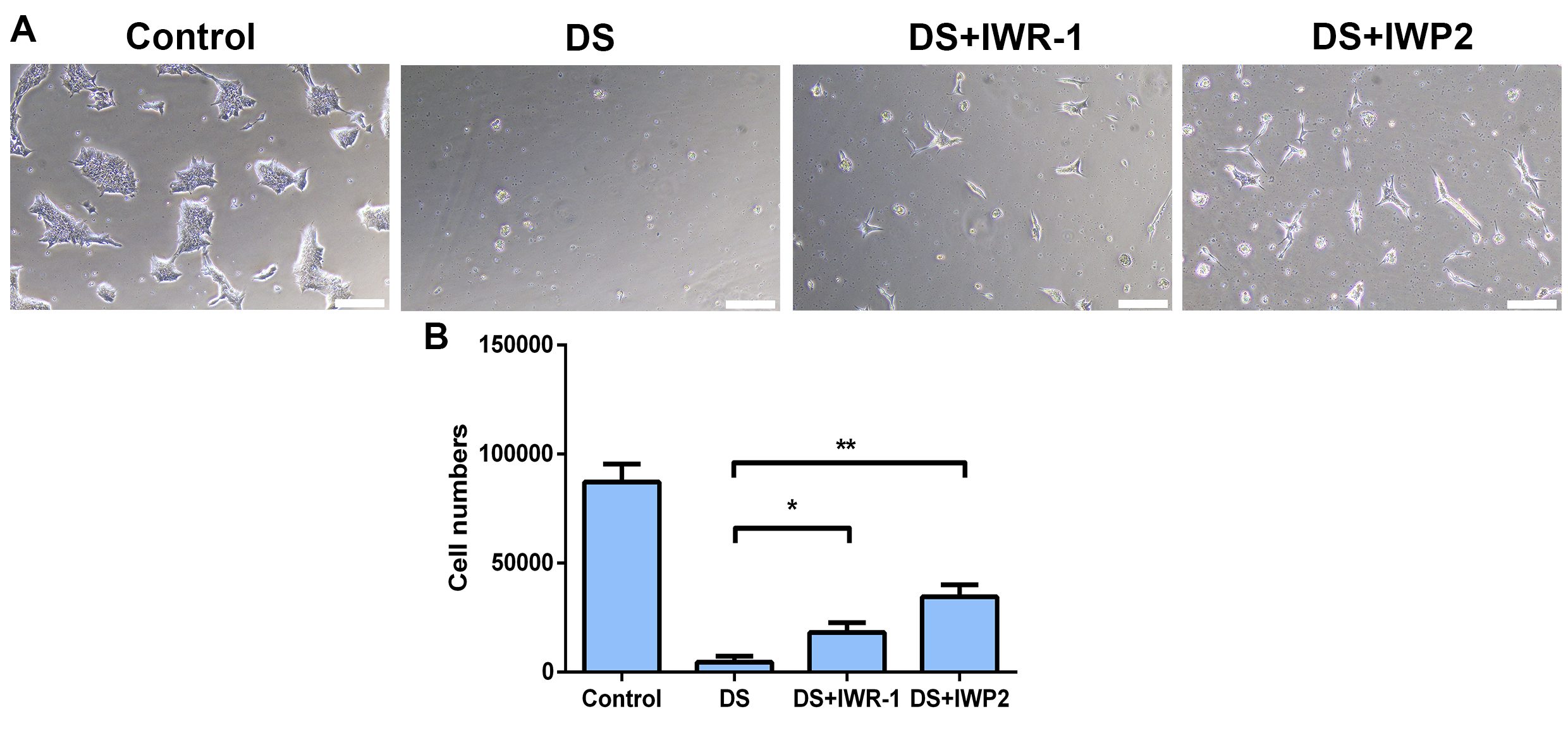

Supplement: Supplementary file 5 — Additional file 5: Fig. S3. Cell counting assay 24 hours after the treatment with DS and Wnt inhibitor. (A) Representative images of the cells treated with or without DS, supplemented with 5 μM Wnt inhibitor IWR-1 or IWP2 24 hours after the treatment. Scale bar = 200 μm. (B) Comparison of the cell numbers 24 hours after inoculation. [file 13287_2022_2890_MOESM5_ESM.tif]

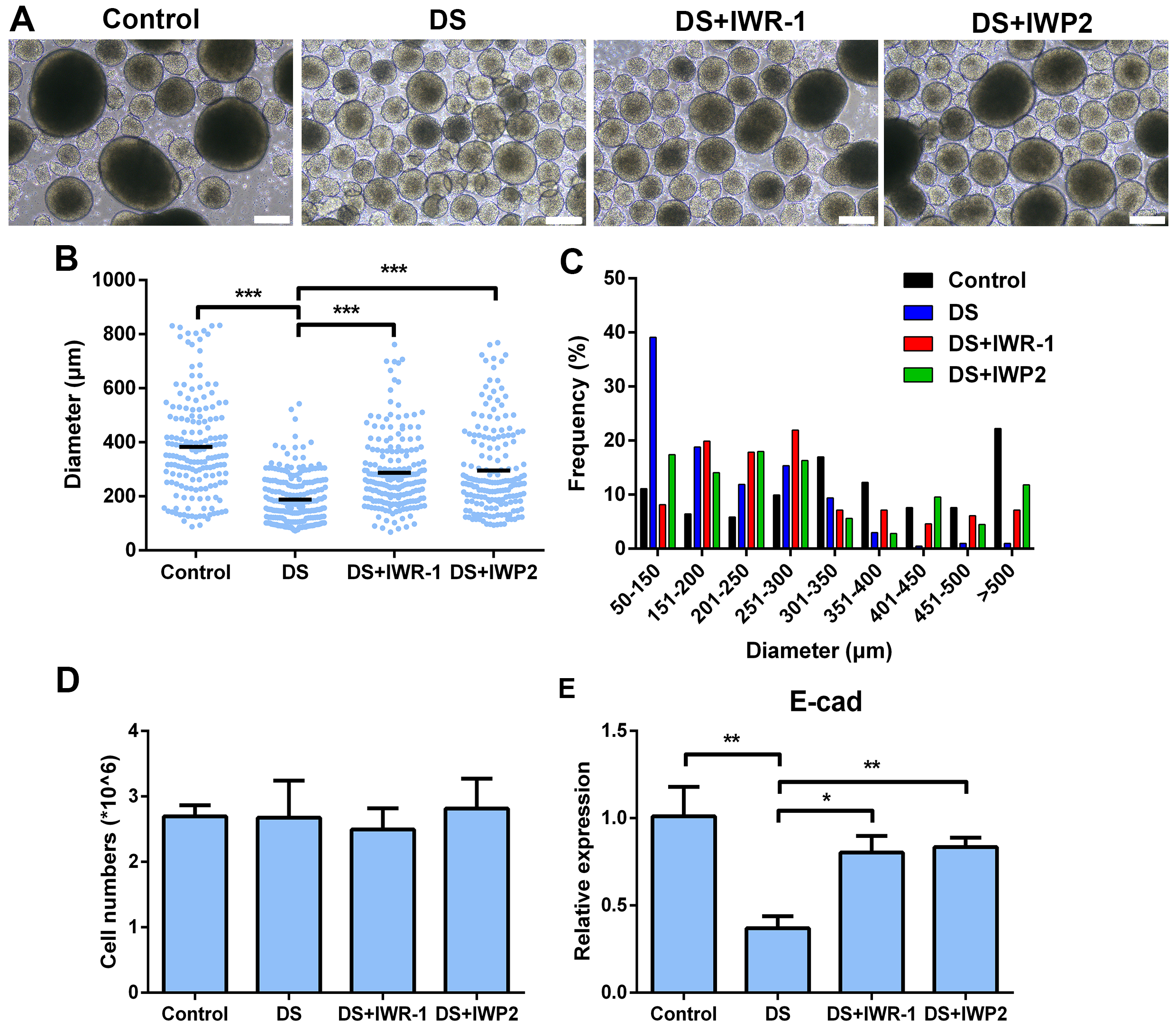

Supplement: Supplementary file 6 — Additional file 6: Fig. S4. Rescue assay with Wnt inhibitors in 3D suspension culture condition. (A) Representative images of the aggregates treated with or without DS plus 5 μM Wnt inhibitor IWR-1 or IWP2 on day 5 after the treatment. Scale bar = 200 μm. (B) Comparison of average diameter of the H9 aggregates on day 5 after the treatment. (C) Diameter distribution of the H9 aggregates on day 5 after the treatment. (D) Comparison of the cell numbers after 5 days of culture. (E) Gene expression analysis by qRT-PCR for E-cad. [file 13287_2022_2890_MOESM6_ESM.tif]

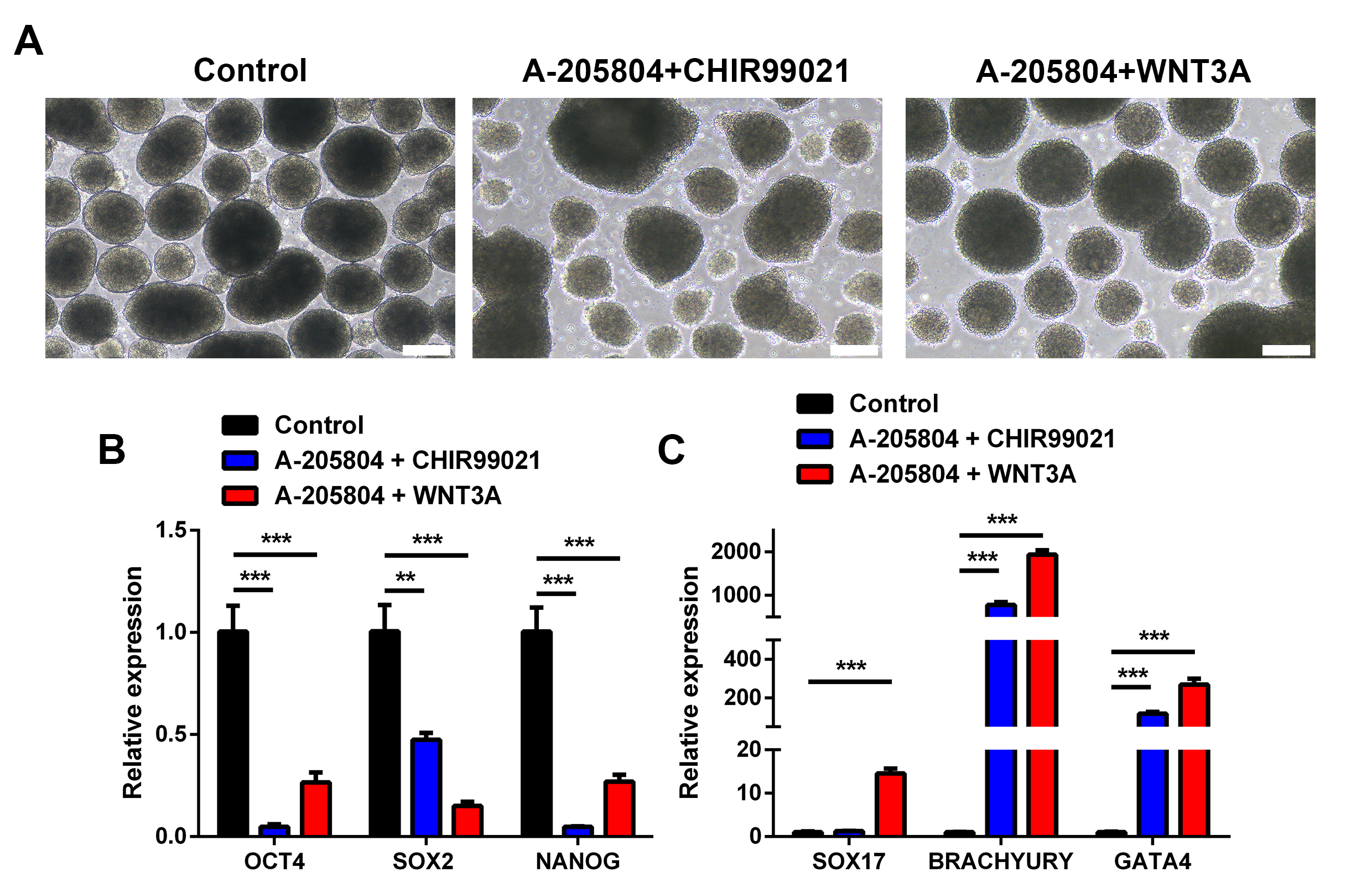

Supplement: Supplementary file 7 — Additional file 7: Fig. S5. Simulation of the effect of DS by replacing DS with ICAM1 inhibitor and Wnt agonist. (A) Representative images of the aggregates treated with or without 10 μM A-205804 plus 3 μM CHIR99021 or 10 ng/ml WNT3A on day 5 after the treatment. Scale bar = 200 μm. (B) Gene expression analysis by qRT-PCR for pluripotent genes, OCT4, SOX2, and NANOG. (C) Gene expression analysis by qRT-PCR for genes SOX17, BRACHYURY, and GATA4 of mesoendoderm germ layers. [file 13287_2022_2890_MOESM7_ESM.tif]
